# Behavioural factors influencing hand hygiene practices across domestic, institutional and public community settings: a systematic review and qualitative meta-synthesis

**DOI:** 10.1136/bmjgh-2025-018927

**Published:** 2025-09-16

**Authors:** Bethany A Caruso, Jedidiah S Snyder, Lilly A O’Brien, Erin LaFon, Kennedy Files, Dewan Muhammad Shoaib, Sridevi K Prasad, Hannah K Rogers, Oliver Cumming, Joanna Esteves Mills, Bruce Gordon, Marlene K Wolfe, Matthew C Freeman

**Affiliations:** 1 Hubert Department of Global Health, Rollins School of Public Health, Emory University, Atlanta, Georgia, USA; 2 Gangarosa Department of Environmental Health, Rollins School of Public Health, Emory University, Atlanta, Georgia, USA; 3 Woodruff Health Sciences Center Library, Emory University, Atlanta, Georgia, USA; 4 Department of Disease Control, London School of Hygiene and Tropical Medicine, London, UK; 5 Water, Sanitation, Hygiene and Health Unit, World Health Organization, Geneva, Switzerland

**Keywords:** Hygiene, Environmental health, Public Health, Systematic review

## Abstract

**Introduction:**

This systematic review sought to understand barriers and enablers to hand hygiene in community settings.

**Methods:**

Eligible studies addressed hand hygiene in a community setting, included a qualitative component, and were published in English between 1 January 1980 and 29 March 2023. Studies were excluded if in healthcare settings or were animal research. We searched PubMed, Web of Science, EMBASE, CINAHL, Global Health, Cochrane Library, Global Index Medicus, Scopus, Public Affairs Information Service Index, WHO Institutional Repository for Information Sharing, UN Digital Library and World Bank eLibrary, manually searched relevant systematic reviews’ reference lists, and consulted experts. We used MaxQDA software to code papers, using the COM-B (Capability, Opportunity, Motivation and Behaviour) framework to classify barriers and enablers. We used thematic analysis to describe each COM-B subtheme identified, GRADE-CERQual to assess confidence in evidence for thematic findings and the Mixed Method Appraisal Tool (MMAT) to assess risk of study bias.

**Results:**

80 studies were included; most took place in Africa (31; 39%), South-East Asia (31; 39%) and domestic settings (54; 68%). The mean MMAT score was 4.86 (good quality). Barriers and/or enablers were reported across all COM-B constructs and subconstructs. The most reported barriers aligned with Physical Opportunity (eg, soap availability), Reflective Motivation (eg, hand hygiene not prioritised) and Automatic Motivation (eg, no habit). In contrast, the most reported enablers aligned with Automatic Motivation (ie, habit) and Reflective Motivation (ie, perception of health risk).

**Conclusion:**

Findings confirm that a lack of necessary resources for hand hygiene hinders practice, even when people are motivated. Results may explain why hand hygiene increases when there are acute health risks (eg, COVID-19), but decreases when risks are perceived to fade. The qualitative methodology used among the studies may have revealed a broader array of barriers and enablers than what might have been found by quantitative, researcher-driven studies, but representativeness may be limited. Evidence was also limited on alcohol-based hand rubs. Findings can inform the design of future hand hygiene initiatives.

**PROSPERO registration number:**

CRD42023429145.

**What is already known on this topic**

- Hand hygiene prevents disease, but barriers like limited access to soap, water and competing priorities hinder practice.
- Most existing reviews focus narrowly on specific behaviors (eg, handwashing with soap and water exclusively) rather than a broad suite of hand hygiene behaviors, or on specific contexts (eg, schools) rather than a broad range of community settings.

**What this study adds**

- This study systematically identifies barriers and enablers to hand hygiene across various community settings using an established behavioural framework.
- Findings highlight the importance of contextual and behavioural factors, showing that resource provision is essential, but alone may not instigate or sustain hand hygiene practices without addressing broader motivational and habitual drivers.

**How this study might affect research, practice or policy**

- Findings from this study can guide the design of targeted interventions that not only ensure the availability of resources but also foster habits and address motivational barriers to hand hygiene.
- Policymakers can leverage these insights to develop more comprehensive hand hygiene programmes, while researchers can explore under-investigated areas such as the barriers and drivers to hand hygiene in community settings among people with disabilities.

## Introduction

Hand hygiene is a critical public health measure, whether performed by washing hands with water and soap or using other methods like alcohol-based hand rubs (ABHR). Hand hygiene interventions, which are relatively inexpensive to implement,^1^ can prevent several infectious diseases, including enteric^2^ and respiratory^3^ infections. These diseases not only account for a large burden of disease globally,^4^ but they also can result in high healthcare costs,^5^ burdening both individuals and health systems. Informed by the profound impact hand hygiene has on health outcomes, guidelines for hand hygiene have been established in healthcare settings,^6^,^9^ and additional guidelines—including those for child health, sanitation, water management and interim COVID-19 guidance—emphasise the importance of investing in hand hygiene as a core public health measure.^10^,^13^

Yet, despite the recognised importance of hand hygiene for health and its cost-effectiveness, there are no global guidelines for hand hygiene in community settings. Community settings are particularly important because they are where people spend most of their time. Specifically, according to the Ottawa Charter, community settings are where “health is created and lived by people within the setting of their everyday life; where they learn, work, play, and love”,^14^ and include domestic, public and institutional spaces.^15^ It is essential to establish global guidelines and tools to implement guideline recommendations for handwashing in these community settings of ‘everyday life’ to best guide hand hygiene initiatives, protect public health and strengthen resilient health systems.^16 17^

There is a specific need for hand hygiene guidance related to behaviour change and determinants of hand hygiene behaviours. A recent scoping review that aimed to identify, summarise and determine the evidence base of existing international hand hygiene guidelines found that the 51 included guidelines largely lacked consistent, evidence-based recommendations and that behaviour change is one of four areas where clear recommendations for hand hygiene in community settings are most needed (other areas included effective hand hygiene; minimum requirements and government measures).^15^ This scoping review also found that existing guidelines provided inconsistent recommendations on what determinants of hand hygiene interventions should target and which, if any, behaviour change models or frameworks should be leveraged.

Understanding what determinants hinder or enable hand hygiene behaviour is necessary for designing appropriate and effective interventions and for assessing if existing interventions appropriately respond to the barriers and enablers identified. Existing systematic reviews on determinants of hand hygiene have been informative but limited in scope. Two recent reviews only included studies that focused on handwashing with soap and water.^18 19^ Other syntheses have focused more narrowly on specific settings, regions and/or countries, including those which only include studies that are in settings likely to include children (eg, schools, homes),^20^ that only focus on hand hygiene practices among primary and secondary school students in sub-Saharan Africa,^21^ and that focus on prevalence and determinants of hand hygiene behaviour in India.^22^ There remains a need for a more comprehensive review to understand the suite of barriers and enablers to a broader range of hand hygiene practices across community settings.

This review seeks to understand the suite of barriers and enablers to hand hygiene in community settings. Intended to support the forthcoming WHO guidelines for hand hygiene in community settings, the research question for this review was identified through an extensive consultation process by the WHO with external experts,^23 24^ following a scoping review of current international guidelines.^15^ Findings from this study can inform intervention design in varied settings to enable improved hand hygiene and reduced transmission of infectious diseases within community settings.

## Methods

### Research questions

This systematic review and qualitative meta-synthesis primarily sought to assess the following question: what are the key behavioural barriers and enablers to practising effective hand hygiene in community settings? See online supplemental file S1 to see the research question in SPIDER format.

In addition, we aimed to understand which theories, models and frameworks have been used in studies investigating barriers and enablers of hand hygiene in community settings and which have been the most common.

### Search strategy

This review was pre-registered with PROSPERO (registration number: CRD42023429145) and is reported in accordance with the Preferred Reporting Items for Systematic Reviews and Meta-Analyses^25^ (PRISMA) criteria (see online supplemental file S2). This review was part of an integrated protocol^24^ for five related reviews to synthesise the evidence for effective hand hygiene in community settings.^26^,^29^ We adopted a two-phased approach for identifying relevant studies. Phase 1 involved a broad search of databases and trial registries to capture all studies on hand hygiene in community settings that were relevant across all five of the systematic reviews. Phase 2 leveraged the reduced sample identified in phase 1, along with additional studies identified through expert consultations and hand-searches of reference lists, to further screen studies (first by title and abstract, then full text review) using criteria specific to this review. A full description of the research questions and the procedures followed for searches, study inclusion, outcome data collection, analysis and reporting of the multiple related reviews is presented in the published protocol.^24^

Briefly, during phase 1, we identified studies published in English or with a title and abstract published in English through late March 2023; the dates of the searches vary by database. Specifically, we searched 12 peer-reviewed and grey literature databases. PubMed, Web of Science, EMBASE (Elsevier), CINAHL (EBSCOhost), Global Health (CAB), Cochrane Library, Global Index Medicus, Scopus (Elsevier), Public Affairs Information Service Index (ProQuest) were searched on 23 March 2023 and WHO Institutional Repository for Information Sharing, UN Digital Library and World Bank eLibrary were searched on 28 March 2023 using search terms related to hand hygiene broadly and with restrictions on terms related to healthcare settings in the titles. We searched trial registries (International Clinical Trials Registry Platform, clinicaltrials.gov) for trials related to hand hygiene in community settings on 29 March 2023.

The Cochrane Library was searched to identify relevant existing reviews. We conducted manual searches of the reference lists of two relevant reviews.^18 19^ For reviews that provided a list of the reviewed articles, we searched only those references. If a list was not available, we searched all references and screened for potentially relevant titles. These reviews had 113 total references, of which 51 were duplicates, 33 were already identified in our database search and 29 were added to phase 2 title and abstract screening. We contacted 35 content experts and organisations, using snowballing methods, between April and May 2023, for information on relevant unpublished literature.

Overall, the outcome of phase 1 was a reduced sample of 6248 records for further screening in phase 2 using criteria specific to this review, including 6216 records from databases and registries (from 47 539) and 32 from hand searching and expert consultation (from 116) (see online supplemental file S3).

### Selection criteria

Studies were eligible for inclusion in this analysis if they addressed barriers or enablers to hand hygiene in community settings, presented qualitative findings, were in English and were published after 1 January 1980. Papers published before 1980 were excluded as they would be over 40 years old and full texts are typically hard to locate. For this review, hand hygiene refers to any hand cleansing undertaken for the purpose of removing or deactivating pathogens from hands and efficacious hand hygiene is defined as any practice which effectively removes or deactivates pathogens from hands and thereby has the potential to limit disease transmission.^7^ The term community settings included domestic (eg, households), public (eg, markets, public transportation hubs, vulnerable populations (eg, people experiencing homelessness), parks, squares or other public outdoor spaces, shops, restaurants and cafes) and institutional (eg, workplace, schools and universities, places of worship, prisons and places of detention, nursing homes and long-term care facilities) spaces.^15^ Studies were excluded if they were in healthcare settings or were animal research. Studies in nursing homes and long-term care facilities were excluded as part of phase 2 screening, as these were determined to be similar to evidence generated in healthcare settings. There were no geographic restrictions.

Studies that presented qualitative findings (ie, qualitative and mixed methods studies) were eligible for inclusion in the analysis. Studies that include qualitative research are best suited to address the research question as they leverage open-ended questions to understand phenomena, perspectives and lived experiences as voiced by participants themselves. In contrast, any barriers or enablers identified in quantitative research will have been pre-identified for investigation based on their inclusion in a survey by the research team. As a result, the survey itself limits the suite of barriers and enablers identified. To report on the scope of research on barriers and enablers of hand hygiene in community settings—regardless of study design—we identified the library of purely quantitative studies focused on enablers and barriers to practising effective hand hygiene in community settings, which we provide as a supplement.

We used Covidence software for systematic reviews.^30^ In both phases, screening of each article (phase 1—title and abstract only; phase 2—title and abstract, then full text review) was performed independently by two reviewers, with discordance between reviewers reconciled by a third reviewer. The stages and related outcomes of the search and screening process are described in the PRISMA flow chart (see online supplemental file S3).

### Data extraction and analysis

For each included study, reviewers independently double extracted data using a customised data extraction tool and independently assessed risk of bias in duplicate using the Mixed Method Appraisal Tool (MMAT).^31 32^ Any conflicts between reviewers were resolved by discussion, which involved collectively reviewing conflicted issues and the study of interest in a meeting. All data extraction tools are provided in online supplemental files S4 and S5.

For appraising the quality of mixed methods studies using the MMAT, the individual study components were assessed using the appropriate categories: the qualitative component, the quantitative category for the quantitative component and the mixed-methods category. Final scores, which can range from 0 to 5 across study types (5 is the best), are presented in the main text of the results only for qualitative components of the MMAT assessment because only qualitative data were extracted from the mixed methods studies included in the review. We provide our grading for both the qualitative and mixed methods components in an online supplemental file.

To provide a broad overview of included studies, we extracted information about study characteristics, including the region, location, setting, participants and hand hygiene practice explored.

To understand which theories, models and frameworks have been leveraged in research investigating barriers and enablers of hand hygiene in community settings and which have been the most common, we extracted the names of any theories, models or frameworks authors reported to have used (if any). We did not extract data on or report how theory was used (eg, in tool design and/or analysis).

To classify the types of barriers and enablers of hand hygiene practices in community settings reported in and across studies and settings, we used the ‘best-fit framework synthesis’ approach,^33^ which has been used in other meta-syntheses related to water, sanitation and hygiene.^34 35^ Following this approach, we identified themes to use as deductive codes a priori from a pre-existing framework. Importantly, the chosen framework does not constrain the analyses; instead, the approach allows for the guiding framework to be iteratively modified on inductive learning from the data, as needed. Specifically, we used the COM-B (Capability, Opportunity, Motivation and Behaviour) framework^36^ as our guide. COM-B has been used to categorise barriers and enablers in multiple systematic reviews,^37^,^39^ including hand hygiene.^20^ The COM-B model proposes that three components, Capability, Opportunity and Motivation, interact to enable and maintain behaviour. Capability can be psychological (knowledge) or physical (skills); opportunity can be social (societal influences) or physical (environmental resources); motivation can be automatic (emotion) or reflective (beliefs, intentions). Table 1 provides comprehensive definitions of each COM-B construct and examples related to hand hygiene.

**Table 1:** Definitions and examples of constructs from the COM-B framework used to code barriers and enablers to practice effective hand hygiene

COM-B construct	Definition*	Examples
**Capability**	**Knowledge, skills and abilities required to engage in a particular behaviour**	
Physical Capability	Physical strength, skill or stamina	Physical ability to wash hands
Psychological Capability	Knowledge/psychological strength, skills or stamina	Knowledge on how to wash hands
**Opportunity**	**External factors which make the execution of a particular behaviour possible**	
Physical Opportunity	Opportunities provided by the environment, such as time, location and resource	Availability of soap
Social Opportunity	Opportunities as a result of social factors, like cultural norms and social cues	Cultural norm to wash hands
**Motivation**	**The internal processes which influence decision-making and behaviours**	
Automatic Motivation	Automatic processes, such as our desires, impulses and inhibitions	Have a habit of washing hands
Reflective Motivation	Reflective processes, such as making plans and evaluating things that have already happened	Believe handwashing reduces illness risk

* Definitions from (Social Change UK).

COM-B, Capability, Opportunity, Motivation and Behaviour.

To identify barriers and enablers in relevant qualitative data, included studies were imported into MAXQDA (V.12; VERBI Software, Berlin, Germany)^40^ qualitative analysis software. Following best practice,^41^ line-by-line coding of the results sections (qualitative sections only for mixed-methods studies), which present the empirical data from the studies, was carried out twice by independent team members. The team developed a codebook to categorise barriers and enablers to practising hand hygiene in community settings (table 1). In addition to the three codes for each COM-B construct (ie, Capability, Opportunity, Motivation) and the six codes for each subconstruct (eg, physical opportunity, social opportunity) identified deductively a priori, we also inductively identified additional thematic codes related to the subconstructs. These inductively created thematic codes were based on team members’ initial reviews of a sample of the included studies and were decided on via discussion. For example, under the ‘physical opportunity’ subconstruct, we inductively created the thematic code ‘affordability’ and three subthematic codes (‘cost of water’, ‘cost of soap’, ‘handwash station maintenance cost’). The codebook was pre-reviewed by one of the creators of the COM-B framework and is provided in online supplemental file S6. Text coded in MAXQDA may have included quotes directly from primary study participants (first-order data) and/or text from authors summarising or synthesising information from their study participants (second-order data).

To assist in aggregating and synthesising findings from the line-by-line coding in MAXQDA, reviewers simultaneously used a customised data extraction tool in Excel (online supplemental file S5) to note if a particular COM-B-related barrier or enabler was identified in each study. Each time a particular barrier or enabler was identified and coded in a study using MAXQDA, the research team member also noted that the specific barrier or enabler was identified on the customised Excel data extraction sheet and cut and pasted the relevant coded text as a means of supporting the coding decision. A third team member not involved in the line-by-line coding then reviewed all codes and copied text segments in the Excel file to confirm that codes were accurately applied, given the copied supporting text.

We identified the frequency with which COM-B-categorised barriers and enablers were identified *across* studies and report these frequencies overall and by setting. We do not report the frequency of coded COM-B-categorised barriers and enablers identified *within* studies because some studies may simply provide more depth on or give additional mention of the same barriers or enablers at different points in the paper based on their analysis strategies. Frequencies, therefore, were only used to report on the extent to which barriers or enablers were identified across studies.

To provide depth on the identified barriers and enablers of hand hygiene practices in community settings categorised using COM-B, we used a thematic analysis approach^42^ to describe each COM-B subtheme identified by setting type and if a barrier or an enabler. We used all coded text related to the subtheme for this analysis, enabling multiple codes from the same study to inform the summary. For example, we examined all codes on the theme of ‘Physical Opportunity: Soap Availability’ to summarise insights from the qualitatively coded data by setting type (domestic, institutional, public) and whether perceived to hinder or enable handwashing. Importantly, for both thematic analysis and tabulation numerically, we aimed to only include the coded text as a barrier or enabler if a distinct connection was made between the subtheme and hand hygiene behaviour. In many instances, studies reported on resources available or absent in a particular setting (eg, soap availability at school), but if the specific text did not report a specific connection between soap availability and whether and how it influenced hand hygiene, it was not included.

To assess the level of confidence in evidence for each thematic finding, we used the GRADE-CERQual approach.^43^ Specifically, for each identified barrier or enabler in a subtheme, we assessed the methodological limitations, coherence, adequacy and relevance by setting (domestic, institutional, public); each criterion was characterised as follows: ‘no or very minor concerns’, ‘minor concerns’, ‘moderate concerns’, ‘serious concerns’. The methodological limitations criteria assessment was informed by the MMAT scores. We leveraged information from the studies involved in each subtheme for the remaining criteria. Specifically, we looked across the coded text that informed the subtheme to assess coherence (fit between primary study data and the synthesised review finding); assessed the number of studies informing the theme to assess adequacy; and reviewed the location, setting, populations engaged and ‘phenomenon of interest’ in included studies to assess relevance.^44^,^47^ Notably, the ‘phenomenon of interest’ for this review and this assessment is hand hygiene broadly, not specific hand hygiene practices (eg, hand washing with soap and water; use of ABHR). All criteria were used to make a final assessment of confidence for each subtheme (high, moderate, low, very low).^48^

### Patient/public involvement

Patients or the public were not involved directly in the design, or conduct, or reporting, or dissemination plans of our research. As noted previously, the research question was developed in broad consultation by the WHO with a network of external experts. Findings from this review will be disseminated alongside the guidelines.

## Results

### Characteristics of the studies included in this review

We identified 80 qualitative and mixed methods studies that met our inclusion criteria; 987 quantitative studies were excluded (onlinesupplemental files S3  S7 and ; the full list of 987 quantitative studies is available along with other relevant data on FigShare). Studies represent all WHO regions, with almost 80% from Africa (31; 39%) and South-East Asia (31; 39%) (table 2). Most studies focused on domestic settings (54; 68%), followed by institutional (21; 26%) and public (15; 19%) settings (totals add up to more than 100% because some studies focused on more than one setting). Within institutional settings, schools were the most studied (14; 67%), with fewer studies in other institutional settings such as workplaces (4; 19%), universities (2; 10%) or childcare centres (1; 5%). Within public settings, internally displaced persons’ camps were most common (7; 47%), followed by markets (5; 33%) and refugee camps (1; 7%). The largest proportion of studies (24; 30%) focused on both men and women for handwashing behaviour; 23% (18) focused on children (boys and girls), 10% (8) targeted women specifically, 7% (5) engaged food workers and 35% (28) focused on the general population or did not specify. Handwashing with soap and water was the most common specified hand hygiene practice of focus (60; 75%); 18% (14) of studies did not provide details about the specific hand hygiene practice of focus. Because only two (2.5%) studies focused on ABHRs, we refer to ‘handwashing’ as the behaviour throughout the results. Regarding specific contexts or ‘risk scenarios’ within which the studies took place, 15% (12) were related to COVID-19 and 10% (8) focused on internal displacement. A total of 987 studies comprises the quantitative library, which is included in the online dataset.^49^

**Table 2:** Characteristics of the included studies

Descriptive characteristics of studies	Total n (%)
Total number of studies	80 (100.0)
Region*	
African region	31 (38.8)
South-East Asian region	31 (38.8)
European region	7 (8.8)
Region of the Americas	7 (8.8)
Eastern Mediterranean region	3 (3.8)
Western Pacific region	2 (2.5)
Location*	
Rural	35 (43.8)
Urban	23 (28.8)
Peri-urban	5 (6.3)
Unspecified	29 (36.3)
Setting*	
Domestic (households)	54 (67.5)
Institutional	21 (26.3)
Schools	14 (66.6)
Workplaces	4 (19.0)
Universities	2 (9.5)
Childcare centres	1 (4.7)
Public	15 (18.8)
Internally displaced persons’ camps	7 (46.6)
Refugee camps	1 (6.6)
Markets	5 (33.3)
Other	2 (13.3)
Study participants*	
Adults—women and men	24 (30.0)
Children—girls and boys	18 (22.5)
Adults—women	8 (10.0)
Food workers	5 (6.3)
Other	5 (6.3)
General population/unspecified	28 (35.0)
Specific hand hygiene practice explored*	
Handwashing with soap and water	60 (75.0)
Handwashing with water only	3 (3.8)
Handwashing with soap alternatives	1 (1.3)
Hand rubbing with alcohol-based rub	2 (2.5)
Handwashing—unspecified	14 (17.5)
Risk scenario	
COVID-19	12 (15.0)
Internal displacement	8 (10.0)
Influenza	1 (1.3)
Typhoid fever	2 (2.5)
HIV	1 (1.3)
Landslide disaster	1 (1.3)
People who inject drugs	1 (1.3)
General emergency setting	1 (1.3)
Study quality rating (mean)†	4.86

* Descriptive statistics for region, location, setting, primary outcome studied, hand hygiene practice and hand hygiene intervention represent variables that were multi-select.

† Quality appraisal using the Mixed Method Appraisal Tool^31 32^: possible scores are 0–5 across study types (5 is the best).

### Quality of the studies included in this review

The mean study quality was 4.86 overall, indicating good quality (5 is the best). The quality appraisal scores for each study are in online supplemental file S8.

### Theory, model or framework used in included studies

A third of the included studies reported use of a theory, model or framework (26; 33%) (table 3). The earliest study included in our review that reported using theory was published in 1994 (online supplemental file S9). 13 different theories, models or frameworks were reported to be used; those most commonly used included Integrated Behavioural Model for Water, Sanitation and Hygiene^50^ (8; 31%), Behaviour Centered Design/Evo-Eco Model^51^ (7; 27%), Health Belief Model^52^ (4; 15%) and COM-B^36^ (4; 15%).

**Table 3:** Theories or models reported to be used in included studies (N=80)

Theory	Total n (%)
Study did not report using theory	**54** (**67.0**)
Study did report using theory*	**26** (**33.0**)
Integrated Behavioural Model for Water, Sanitation, and Hygiene (IBM-WASH)	8 (30.8)
Behaviour Centered Design (BCD)/Evo-Eco Model	7 (26.9)
Health Belief Model (HBM)	4 (15.4)
Capability, Opportunity, Motivation and Behaviour (COM-B)	4 (15.4)
Risks, Attitudes, Norms, Abilities and Self-regulation (RANAS)	2 (7.7)
Theory of Planned Behaviour (TPB)	2 (7.7)
Theoretical Domains Framework	2 (7.7)
Elicitation Procedure	1 (3.8)
Cultural Consensus Theory	1 (3.8)
Prototype Theory	1 (3.8)
Donabedian’s Model for Assessing Quality of Care	1 (3.8)
PRECEDE-PROCEED model	1 (3.8)
The Model of Families’ Use of Health Services	1 (3.8)

* Multiple theories could have been reported per study, so the sum of theories used (35) is greater than the total number of studies that report using theory (26). Specifically, one study used Behaviour Centered Design (BCD)/Evo-Eco Model, RANAS and HBM; one study used Behaviour Centered Design (BCD)/Evo-Eco Model and COM-B; one study used Behaviour Centered Design (BCD)/Evo-Eco Model and IBM-WASH; one study used IBM-WASH and the PRECEDE-PROCEED model; one study used HBM and TPB; one study used HBM and The Model of Families Use of Health Service; and one study used Cultural Consensus Theory and Prototype Theory. Additionally, see online supplemental file S7 to see which study used which theories.

### COM-B-categorised barriers and enablers of hand hygiene practices across community settings

**Figure 1:**
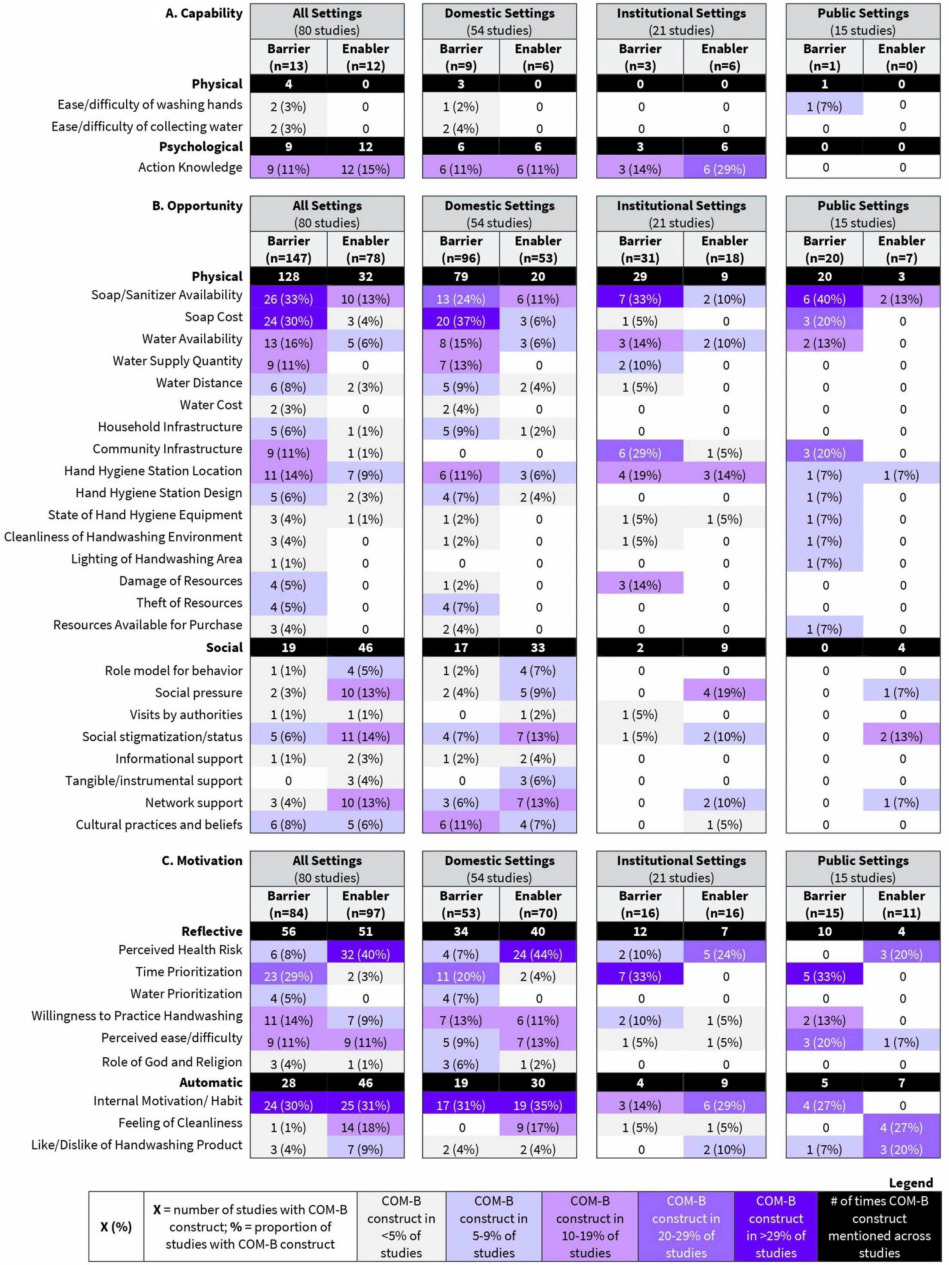
Frequency of participant-reported barriers and enablers to hand hygiene practices in community settings categorised by (**A**) Capability, (**B**) Opportunity and (**C**) Motivation components of the Capability, Opportunity, Motivation and Behaviour (COM-B) framework.

Across all settings, barriers and/or enablers were reported across all COM-B constructs and subconstructs, though some constructs were more frequently reported as barriers while others were more frequently reported as enablers (figure 1). Specifically, the most reported barriers overall and across settings aligned with *Physical Opportunities* (ie, ‘soap availability’, ‘cost of soap’, ‘water availability’), *Reflective Motivation* (ie, ‘time prioritisation’) and *Automatic Motivation* (ie, ‘internal motivation/habit’). Several subthemes of water—related to *Physical Opportunity*—were also noted as barriers for different reasons and with varied frequency. If assessed in combination, water also emerges as a substantial barrier (‘water availability’ (13/80, 16%); ‘water supply quantity’ (9/80, 11%); ‘water distance’ (6/80, 7%); all water collectively (28/80, 35%)). In contrast, the most reported enablers aligned with *Reflective Motivation* (ie, ‘disease risk’) and *Automatic Motivation* (ie, ‘internal motivation/habit’).

Beyond the frequencies of reported barriers and enablers, brief summaries of each subtheme were created from a close reading of the qualitative findings and are further referenced in the sections that follow. A table of thematic summaries is provided in an online supplemental file, disaggregated by whether or not the subtheme is noted to be a barrier or enabler (online supplemental file S10). These thematic summaries are sorted by the COM-B framework and presented by setting type. The table also notes the specific sources that inform the summaries as well as the overall confidence in the evidence for each subtheme (ie, GRADE-CERQual rating). Justifications for the provided GRADE-CERQual ratings are provided in the final online supplemental file S11. Despite the review focussing on hand hygiene broadly, most studies (60; 75%) reported on handwashing with water and soap and thus the summaries largely reflect this specific practice.

### COM-B-categorised barriers and enablers of hand hygiene practices in domestic settings

In domestic settings, the most reported barriers were related to *Physical Opportunity* (‘cost of soap’ (20/54; 36%) and ‘soap availability’ (13/54; 24%)), *Automatic Motivation* (‘internal motivation/habit’ (17/54; 31%)) and *Reflective Motivation* (‘time prioritisation’ (11/54; 20%)). Further, if assessed together, water-related barriers were a commonly noted barrier in domestic settings (‘water availability’ (8/54, 15%); ‘water supply quantity’ (7/54, 13%); ‘water distance’ (5/54, 9%); all water collectively (20/54, 37%)).

Based on thematic analysis (online supplemental file S10), studies found that the lack of soap available in domestic settings and the cost of soap (both *Physical Opportunity*) hindered handwashing practices. Confidence in these themes, based on the GRADE-CERQual Assessment, was high (online supplemental file S11). Regarding water-related themes, studies described both a lack of water and an irregular water supply as hindrances. Distance to water sources hindered handwashing altogether or required people to limit/ration the amount of water people used for handwashing, if practised (confidence in these water themes was moderate and high).

Regarding ‘internal motivation/habit’ (*Automatic Motivation*; high confidence), study participants noted that they lacked a routine or habit for practising hand hygiene; they specifically said they would forget or not wash hands due to ‘laziness’ or noted that they had other habits or routines already established that did not include handwashing, thus hindering practice. Related to ‘time prioritisation’ (*Reflective Motivation*; high confidence), studies discussed that the amount of time required for handwashing prevented them from the behaviour altogether or handwashing with soap specifically. Handwashing was described as inconvenient and less of a priority for their time over other activities that need their attention, particularly for mothers (eg, preparing and serving food, childcare and other general household responsibilities).

The most commonly reported enablers in domestic settings related to *Reflective* (‘perceived health risk’ (24/54; 44%)) and *Automatic Motivation* (‘internal motivation/habit’ (19/54, 35%)). Study findings show that when people identified a risk to their health, they were motivated to start, maintain or increase the frequency with which they practise handwashing. Similarly, perceiving handwashing to be good for health also influenced the behaviour (high confidence). Regarding ‘internal motivation/habit’ studies show that an established habit, routine or perceived ‘need’ to wash hands influences practice, and that these routines, habits and needs were often described in relation to other preceding activities (like toileting) or feelings (disgust).

Additional information describing how and why other subthemes are noted to be barriers and enablers in the domestic setting is provided in online supplemental S10.

### COM-B-categorised barriers and enablers of hand hygiene practices in institutional settings

In institutional settings, ‘soap availability’ (*Physical Opportunity*; 7/21, 33%), ‘time prioritisation’ (*Reflective Motivation*; 7/15, 33%) and ‘community infrastructure’ (*Physical Opportunity*; 6/21, 29%) were the most frequently reported barriers. As in domestic settings, studies found that the lack of soap availability in institutional settings hindered handwashing practice (high confidence). ‘Time prioritisation’, specifically time pressure, being busy and having competing priorities or interests—for example, kids preferring to use time to play over waiting in a queue while at school—also limited handwashing (high confidence). Finally, both a lack of infrastructure or having limited access to infrastructure in institutional settings were described to hinder behaviour (high confidence).

The most commonly reported enablers in institutional settings were ‘internal motivation/habit’ (*Automatic Motivation*; 6/21, 29%), ‘action knowledge’ (*Psychological Capability*; 6/21, 29%) and ‘perceived health risk’ (*Reflective Motivation*; 5/21, 24%). The presence of a habit was described as driving handwashing habits in institutional settings, though studies were limited in both geographic (half in the USA) and setting scope (mostly schools) (moderate confidence). ‘Action knowledge’, specifically practical knowledge about when and how to wash hands was reported in studies to influence handwashing, though studies were mostly in education settings (schools, universities) and had limited geographic scope (moderate confidence). Finally, regarding ‘perceived health risk’, studies reported handwashing behaviour to be influenced both by whether individuals perceived their health to be at risk or the health of others (eg, customers in a workplace setting) to be at risk if they do not wash their hands. The theme is predominantly informed by studies in Africa and in education settings (moderate confidence).

Additional information describing how and why other subthemes are noted to be barriers and enablers in institutional settings is provided in online supplemental file S10

### COM-B-categorised barriers and enablers of hand hygiene practices in public settings

In public settings, the most frequently reported barriers were ‘soap availability’ (*Physical Opportunity*; 6/15, 40%), ‘time prioritisation’ (*Reflective Motivation*; 5/15, 33%) and ‘internal motivation/habit’ (*Automatic Motivation*; 4/15, 27%). As with both domestic and institutional settings, lack of access to soap was found to hinder handwashing practice, though most of the small number of studies (5 of 6) were in Africa, of which half were focused on IDP camps, limiting information from other geographies and public settings (moderate confidence). Regarding ‘time prioritisation’, being busy or having competing priorities or interests was noted to hinder handwashing at all or as noted in guidelines. Specifically, one study noted that participants were reluctant to follow guidelines because they felt the process to be prolonged if they were to rub and use soap as directed (moderate confidence). Finally, in terms of ‘internal motivation’ participants’ cited their handwashing to be hindered by a lack of habit, routine or ability to remember to wash their hands, as well as the presence of other routines that do not include handwashing. A participant from one study at a harm reduction centre in France summarised how age can also be a factor in whether or not habits can evolve: “it’s good for the new generations who are going to adopt this practice. It’s more complicated for older generations”.^53^

The most common enablers in public settings were ‘feeling of cleanliness’ (*Automatic Motivation*; 4/15, 27%), ‘like of handwashing product’ (*Automatic Motivation*; 3/15, 14%) and ‘perceived health risk’ (*Reflective Motivation*; 3/15, 14%). While these were the most common enablers, confidence in evidence for the themes was either low or very low; the limited number of studies contributed to lack of coherence, adequacy and relevance. Regarding ‘feeling of cleanliness’, included studies reported handwashing being motivated because the practice made participants feel clean and refreshed, and for ‘like of handwashing product’, the feeling of the product (eg, not greasy), the smell and how it made hands feel (eg, feel soft, smooth), inspired use. Finally, as in other settings, illness prevention was reported to drive handwashing.

Additional information describing how and why other subthemes are noted to be barriers and enablers in public settings is provided in online supplemental file S10.

## Discussion

Our review of hand hygiene barriers in community settings relied on qualitative data to ensure understanding of the factors that influence behaviour from individual perspectives. We found handwashing with soap and water to be the most dominant hand hygiene behaviour assessed. Most of the research took place in Africa and Southeast Asia and in domestic settings and in rural areas, with limited research in workplaces and public spaces. No studies were identified that focused on persons with disabilities. Notably, across all settings, a lack of soap and water (‘Physical Opportunity’) was a dominant barrier, underscoring how vital these resources are to practising hand hygiene. Even where water and soap were available, constrained access to these resources, including high perceived costs of soap or distance to water sources, was reported to impact the regular practice of hand hygiene. ‘Perceived health risk’ and ‘time prioritisation’ (‘Reflective Motivation’) and the presence (or absence) of a habit (‘Automatic Motivation’) are other highly reported barriers/enablers that should be considered, based on context, once ensuring that water, soap or other enhancements to the physical environment are in place. We discuss these key findings related to the dominant barriers and enablers identified across settings.

### Access to soap and water is essential, but alone likely insufficient to motivate or sustain hand hygiene

Consistent with other reviews,^18 19^ our findings confirm that a lack of an enabling physical environment—that is, one that provides the resources necessary for hand hygiene like water and soap—can hinder hand hygiene behaviours. Specifically, a 2023 systematic review, which sought to determine the barriers and enablers specific to handwashing with soap in community settings (46 studies), found ‘environmental context and resources’ to be the most common domain described. The authors’ description of this domain aligns with what is referred to in this review as ‘Physical Opportunity’. (Like ‘Physical Opportunity’, the 2023 review authors note that their ‘environmental context and resources’ domain includes a lack of handwashing resources, like handwashing station hardware, water and soap.^19^) Collectively, these findings underscore the imperative for people to have access to water and soap and/or ABHRs to practise hand hygiene.

Even beyond the sphere of hand hygiene, making changes to environments is understood to be crucial to facilitating positive behaviours. Specifically, interventions that change the environmental context not only ‘require less individual effort and have the greatest population impact’ but also help to make the behaviour a ‘default choice’.^54^ Yet, despite the obvious need for access to soap and water (or other hand hygiene materials like ABHRs), another recent review found that interventions designed to improve hand hygiene in community settings are not always providing these necessary resources. Instead, interventions largely focused on providing instructions on how to wash hands (an example of ‘action knowledge’ under ‘Psychological Capability’).^28^ In our review, ‘Psychological Capability’ was not a dominant barrier or enabler, except in school settings where ‘action knowledge’ was noted as an enabler, particularly among school children who may have been learning how to perform the behaviour correctly for the first time. Critically, initiatives that aim to improve hand hygiene in community settings—whether in domestic, institutional or public spaces—first need to enhance the environment with the resources required for handwashing or ensure that they are already in place either before or simultaneously to addressing other barriers or enablers. Those designing such initiatives should consider developing a theory of change and/or carrying out problem tree analyses to map how the proposed programmes are expected to enable hand hygiene behaviour change.^55 56^

### Motivation influences hand hygiene and needs to be considered alongside resource provision

A ‘Reflective Motivation’ theme, ‘time prioritisation’, was found to hinder behaviour; similar concerns about not having time for handwashing or deeming other activities to be priorities were also noted in other reviews.^18 19^ Specifically, when individuals had competing priorities, interests or needs, or felt that handwashing—or accessing the materials necessary for handwashing—was too time consuming, they were deterred from practising the behaviour. Following from what was noted previously, individuals may feel that handwashing is not ‘as good’ or ‘as important’ as other behaviours or practices that require their limited time and attention. As such, in addition to elevating what is ‘good’ about handwashing, initiatives can consider how to improve self-efficacy so that handwashing is not believed to be a challenging, time-consuming task in competition with other behaviours. Initiatives should ensure that resources are not just available in the environment but easily accessible and conveniently located to minimise or eliminate the time burden and individuals’ associated beliefs that handwashing is inconvenient and they are not able to perform it. Lessons can be learnt from research in healthcare settings, which has found that increasing accessibility and visibility of hand hygiene stations improved handwashing behaviours.^57 58^ Additionally, interventions could ‘bundle’ hand hygiene *with* relevant priority behaviours, so it is considered a fundamental component of key tasks they would not compromise. For example, interventions have effectively bundled handwashing with child feeding into a ‘mealtime’ package, so individuals do not see handwashing as separate from feeding their children, but as a part of the feeding routine.^59^

### Building habits is key to sustaining hand hygiene behaviours, provided hygiene resources are available

When people perceived there to be a health risk, they were driven to start handwashing, maintain their practice or increase the frequency of existing handwashing behaviours. This other ‘Reflective Motivation’ finding may help explain variation in handwashing behaviour when health risks were perceived to be more or less acute. For example, a study involving over 6000 adults across 14 countries on five continents found there to be higher levels of handwashing adherence when COVID-19 cases were on the rise in the previous 2 weeks. Conversely, handwashing fell when COVID-19 cases were not noticeably on the rise, or ‘salient’, in the previous 2 weeks, but were still gradually accumulating.^60^ The authors observed that this ‘falling and peaking’ pattern of handwashing behaviour continued depending on the trajectory of the virus, and that people did not experience a ‘pandemic fatigue’ that eventually led them to stop washing hands and never start up again. During the ‘fall’ part of the cycle, individuals may have lost the belief or perception that the health risk was acute, leading them to believe that handwashing was no longer an urgent need. Important, though, is that the authors found that the cycle would ‘peak’ again when the perception of risk became more salient, during which time people would resume more adherent handwashing behaviours. As it would not be advisable to try to maintain people’s concern about the intensity of acute health risks simply to drive handwashing behaviour, interventions can work to enshrine the belief or judgement that adherence to handwashing is a ‘good’ behaviour even when risks are less acute.

The theme ‘internal motivation/habit’ (Automatic Motivation) was the only subtheme identified as a dominant barrier and enabler both overall and in domestic and institutional settings. Across various studies, individuals reported how handwashing was easy because they already had a habit or that it was not something they did because they lacked a habit. Different approaches are needed to address ‘Automatic Motivation’ depending on whether habits need to be created or maintained. Establishing new routines and regularly practising or repeating these routines until they are automatic is seen as critical for habit formation,^61^ as is adding cues to the environment to trigger the behaviour or ‘piggy-backing’ on other established routines.^62^ Critically, effort should be made to maintain environments that are supportive of existing habits; when environments that previously supported habits change, habits might be broken.^62^ Specifically, if hand hygiene materials are consistently available to encourage handwashing or sanitising but then are removed, people may not maintain the habit by seeking out or providing the materials for themselves. In fact, changing the environment, which can disrupt an individual’s routine, is seen as a strategy for breaking bad habits.^62^ Therefore, an unintentional outcome of no longer providing hand sanitisers in public spaces like schools, transport hubs or shops—as was typical during the height of the COVID-19 pandemic—is that people will no longer practise hand hygiene when entering through a door to a new space. Even worse, the absence of a hand hygiene station may act as its own cue to signal that the behaviour is no longer necessary.

Findings underscore the importance of not only establishing habits for hand hygiene but ensuring that habits can be maintained by ensuring that necessary resources are available to enable it (eg, creating ‘Physical Opportunity’). If resources are removed or are not replenished when needed, behaviours that were well established may be easily forgotten. Further, many studies discussed how people had habits that did not include hand hygiene, or their habit was not to practise hand hygiene. As such, in many cases, interventions and programmes may need to not only aim to create habits, but they also may need to break existing habits that do not include hand hygiene.

### Hand hygiene interventions must be inclusive, addressing the barriers and enablers for all

In our review, no studies focused on barriers and enablers of hand hygiene practice for people with disabilities. People with disabilities are a key vulnerable group that faces specific barriers hindering their hand hygiene practice, with barriers varying by the type of disability.^63^ A study during the COVID-19 pandemic arosss five different countries (Kenya, Indonesia, Zambia, Sierra Leone and Bangladesh) shows that people with disabilities faced significant barriers to accessing and using handwashing stations in both household and public settings.^64^ Understanding the varied barriers and enablers for people with disabilities is necessary to ensure that interventions are adequately designed to improve hand hygiene for all.

### Strengths and limitations

This review was part of an integrated protocol for multiple related reviews, which included an exhaustive search strategy encompassing multiple databases and grey literature sources, and a two-phased approach to identify relevant literature of hand hygiene in community settings. To understand barriers and enablers, we deliberately sought qualitative research. Qualitative approaches encourage those engaged to voice their own insights and ideas in an open-ended way. Therefore, we expected that the studies included may have captured a broader array of insights regarding hand hygiene behaviour than what would have been captured by quantitative studies, which would use researcher-developed surveys to assess the frequency or agreement of barriers and enablers selected a priori. However, to carry out an analysis of qualitative findings, we excluded articles published before 1980 as they are typically hard to locate, and we only included studies that were published in English. It is possible that we missed critical insights from older studies and from studies published in other languages; the exclusion of research published in Spanish may explain the low number of studies in the region of the Americas.

In reviewing and assessing the barriers and enablers described in the studies, we applied the widely used COM-B framework. While well-regarded in behaviour change research, the COM-B model was only used by 5% of the studies included and our categorisation of behavioural barriers and enablers may be different from what the primary study authors might have done. In addition, the use of COM-B over other frameworks may have prevented us from investigating other phenomena that emerged from the data, such as issues around equity. Still, we are confident that the COM-B framework was well-suited for the primary research question as it enabled us to compare findings and identify trends across diverse studies. Further, while we did investigate the use of theory among included studies, this study aimed only to see how common it was for authors to report the use of theory in research on hand hygiene in community settings. It was beyond the scope of this study to understand *how* theory was used (eg, in design of tools, during analysis, etc) and if done so effectively; further research could be undertaken to explore how theory was used (e.g., in design of tools, during analysis, etc.).

While this review did not focus solely on handwashing with water and soap, only a limited number of studies (six) explicitly investigated barriers and enablers to hand hygiene with other materials (eg, water only, soap alternatives and alcohol-based rub). Because of this limited sample, we are not able to discern if barriers and enablers are different based on hand hygiene methods and encourage further research, particularly into ABHRs which are widely used and were ubiquitous during the height of the COVID-19 pandemic.

A final limitation of this review is that our search date exceeds 12 months. However, this ‘one-size-fits-all’ timeline recommendation has been argued to hinder the dissemination of important research, particularly for broad, complex and multicomponent reviews, like this one, which tend to take longer to complete, age more slowly and are less likely to become outdated.^65^ Notably, this review was part of an integrated protocol covering five systematic reviews and 22 research questions, with a comprehensive, two-phased search approach to capture relevant studies on hand hygiene in community settings. These reviews required significant coordination, conceptual development and interpretation, and the qualitative analyses for this review involved multiple synthesis stages. The results for this review and an initial manuscript draft were provided to the WHO team by March 2024 to feed into the ongoing guideline work. The timeline to publication was further extended after results were finalised to share with the WHO and experts engaged in the development of forthcoming guidelines, ensuring the findings and interpretation were relevant and applicable, enhancing the review’s utility.

## Conclusion

Our finding that a lack of access to soap and water was a dominant barrier to hand hygiene but that access to these resources was not a major enabler suggests that soap and water are necessary but likely insufficient to drive hand hygiene behaviour, depending on context and circumstances. As such, programmes that seek to enable hand hygiene behaviours should first ensure that physical environments are equipped with the resources necessary for handwashing and then look to other behavioural enablers and barriers, with attention to the specific setting and the local context.

## Supplementary material

10.1136/bmjgh-2025-018927 online supplemental file 1

10.1136/bmjgh-2025-018927 online supplemental file 2

10.1136/bmjgh-2025-018927 online supplemental file 3

10.1136/bmjgh-2025-018927 online supplemental file 4

10.1136/bmjgh-2025-018927 online supplemental file 5

10.1136/bmjgh-2025-018927 online supplemental file 6

10.1136/bmjgh-2025-018927 online supplemental file 7

10.1136/bmjgh-2025-018927 online supplemental file 8

10.1136/bmjgh-2025-018927 online supplemental file 9

10.1136/bmjgh-2025-018927 online supplemental file 10

10.1136/bmjgh-2025-018927 online supplemental file 11

## Data Availability

Data are available in a public, open access repository. All data relevant to the study are included in the article or uploaded as supplementary information.
