# Supplementary material for: Behavioural factors influencing hand hygiene practices across domestic, institutional and public community settings: a systematic review and qualitative meta-synthesis
Source: BMJ Glob Health. 2025 Sep 16;10(Suppl 7):e018927. doi: 10.1136/bmjgh-2025-018927 (PMC12443170; doi:10.1136/bmjgh-2025-018927)
Supplement: online supplemental file 1 [file bmjgh-10-Suppl_7-s001.docx]

**Behavioural factors influencing hand hygiene practices across domestic, institutional, and public community settings: A systematic review and qualitative meta-synthesis**

Bethany A. Caruso^1^, Jedidiah S. Snyder^2^, Lilly A. O’Brien^2^, Erin LaFon^2^ , Kennedy Files^2^, Dewan Muhammad Shoaib^1^, Sridevi K. Prasad^1^ , Hannah Rogers^3^ , Oliver Cumming^4,5^, Joanna Esteves Mills^5^, Bruce Gordon ^5^, Marlene K. Wolfe^2*^, Matthew C. Freeman^2*^

1 Hubert Department of Global Health, Rollins School of Public Health, Emory University, Atlanta, GA, USA;

2 Gangarosa Department of Environmental Health, Rollins School of Public Health, Emory University, Atlanta, GA, USA;

3 Woodruff Health Sciences Center Library, Emory University, Atlanta, GA, USA;

4 Department of Disease Control, London School of Hygiene and Tropical Medicine, London, UK;

5 Water, Sanitation, Hygiene and Health Unit, World Health Organization, Geneva, Switzerland.

Corresponding author: Bethany A. Caruso; bcaruso@emory.edu

Emory University, Rollins School of Public Health, 1518 Clifton Rd, Atlanta, GA 30322

*Contributed equally.

**Table S1.** Research question and eligibility criteria in SPIDER format.

| **Research Question** | **Sample** | **Phenomenon of Interest** | **Design** | **Evaluation** | **Research Type** |
| --- | --- | --- | --- | --- | --- |
| What are key behavioural barriers and enablers to practicing effective hand hygiene in community settings? | General population in community settings | Behavioral barriers and enablers for practicing hand hygiene | Phenomenology, barrier analysis, grounded theory, thematic analyses, cross-sectional/ observational | Effective hand hygiene (ie, any practice which removes or deactivates pathogens from hands and thereby limits diseases transmission) | Qualitative,  Mixed methods (only qualitative portion used) |
