# Supplementary material for: Behavioural factors influencing hand hygiene practices across domestic, institutional and public community settings: a systematic review and qualitative meta-synthesis
Source: BMJ Glob Health. 2025 Sep 16;10(Suppl 7):e018927. doi: 10.1136/bmjgh-2025-018927 (PMC12443170; doi:10.1136/bmjgh-2025-018927)
Supplement: online supplemental file 4 [file bmjgh-10-Suppl_7-s004.docx]

**Behavioural factors influencing hand hygiene practices across domestic, institutional, and public community settings: A systematic review and qualitative meta-synthesis**

Bethany A. Caruso^1^, Jedidiah S. Snyder^2^, Lilly A. O’Brien^2^, Erin LaFon^2^, Kennedy Files^2^, Dewan Muhammad Shoaib^1^, Sridevi K. Prasad^1^, Hannah Rogers^3^ , Oliver Cumming^4,5^, Joanna Esteves Mills^5^, Bruce Gordon ^5^, Marlene K. Wolfe^2*^, Matthew C. Freeman^2*^

1 Hubert Department of Global Health, Rollins School of Public Health, Emory University, Atlanta, GA, USA;

2 Gangarosa Department of Environmental Health, Rollins School of Public Health, Emory University, Atlanta, GA, USA;

3 Woodruff Health Sciences Center Library, Emory University, Atlanta, GA, USA;

4 Department of Disease Control, London School of Hygiene and Tropical Medicine, London, UK;

5 Water, Sanitation, Hygiene and Health Unit, World Health Organization, Geneva, Switzerland.

Corresponding author: Bethany A. Caruso; bcaruso@emory.edu

Emory University, Rollins School of Public Health, 1518 Clifton Rd, Atlanta, GA 30322

*Contributed equally.

# Extraction fields for RQ3.1: Barriers and Enablers to Hand Hygiene in Community Settings

|  | Item extracted in all RQs |
| --- | --- |
|  | Item specific to Barriers and enablers (3.1) |

| **#** | **Field** | **Details** | **Entry** |
| --- | --- | --- | --- |
| **1. Information about the study** | | | |
| 1.1 | Study design |  | *Select one*  (1) Qualitative study  (2) Mixed methods  (3) Non-comparative  (4) Experimental Before-and-after study  (5) Observational Before-and-after study  (6) Experimental Times series  (7) Observational Times series  (8) Non-randomized trial  (9) Non-randomized cross-over design  (10) Randomized controlled trial  (11) Randomized cross-over study  (12) Analytical cross-sectional study  (13) Case-control study  (14) Cohort study  (777) Other – specify |
| 1.2 | Registered trial | Does the study report that it is linked to a registered trial? (e.g., clinicaltrials.gov, ICTRP) | *Select one*  (1) Yes  (0) No |
| 1.3 | Trial # | If linked to a registered trial, paste trial registration number | Text  (999) Not applicable |

| **#** | **Field** | **Details** | **Entry** |
| --- | --- | --- | --- |
| **2. Eligibility** | | | |
| 2.1 | Confirm participants/sample | Does the study include general populations in community settings?  DO NOT PROCEED IF “NO” | *Select one*  (1) Yes  (0) No |
| 2.2 | Confirm phenomena of interest | Is the phenomena of interest “behavioral barriers and enablers for practicing hand hygiene?”  DO NOT PROCEED IF “NO” | *Select one*  (1) Yes  (0) No |
| 2.3 | Confirm study design | Is the design of the study NOT a quantitative non-comparative study?  DO NOT PROCEED IF “NO” | *Select one*  (1) Yes  (0) No |
| 2.4 | Confirm evaluation | Does the study include the evaluation of hand hygiene practice (i.e., any action of hand cleansing for the purpose of removing or deactivating pathogens from hands)?  DO NOT PROCEED IF “NO”~~’~~ | *Select one*  (1) Yes  (0) No |
| 2.5 | Confirm research type | What research type does the study use?  DO NOT PROCEED IF NONE ARE CHECKED | *Select one*  (1) Qualitative  (2) Quantitative  (3) Mixed methods |

| **#** | **Field** | **Details** | **Entry** |
| --- | --- | --- | --- |
| **3. Setting** | | | |
| 3.1 | Country | Which country is represented in the study? (List all countries separated by a comma, if study is from multiple sites) | Text  999 = Not applicable |
| 3.2 | Region | Which region is represented in the study? | *Check multiple*  (1) Africa  (2) Asia  (3) Europe  (4) Latin America/Caribbean  (5) Middle East  (6) North America  (8) Oceania  (10) Unspecified  (999) Not applicable |
| 3.3 | Urban/Rural | Does the setting of the population fall under any of these specific categories? Select all that apply | *Check multiple*  (1) Urban  (2) Rural  (3) Peri-urban  (777) Other - specify  (888) Not reported |
| 3.4 | Community setting | Does the setting of the population fall under any of these specific categories? Select all that apply | *Check multiple*  (1) Domestic - Households  (2) Public - Markets  (3) Public - Public transportation hubs  (4) Public - Parks, squares, or other public outdoor spaces,  (5) Institutions - Workplace  (6) Institutions - Schools  (7) Institutions - Universities  (8) Institutions - Places of worship  (9) Institutions - Prisons and places of detention  (10) Internally displaced people camps  (777) Other - specify  (888) Not reported |

| **#** | **Field** | **Details** | **Entry** |
| --- | --- | --- | --- |
| 3.5 | Risk scenarios | Did the study evaluate hand hygiene in a risk scenario? | *Select one*  (0) No  (1) Yes  (999) Unclear |
| 3.6 | Risk scenario type | If yes, what was the risk scenario? | *Select one*  (1) COVID-19  (2) Flu  (3) Earthquake  (4) Flood  (5) Typhoon  (6) Forced migration  (7) Internal displacement  (8) Emergency setting  (777) Other – specify  (999) Not applicable |
| 3.7 | Risk scenario text | Please copy in the author’s text about the risk scenario. | Text (999) Not applicable |
| **4. Methods** | | | |
| 4.1 | Aim of study | Copy and paste the aim/ objective/ purpose/goal as stated in the study | Text  (888) Not reported |
| 4.2 | Primary study outcome | What was the primary outcome for this study?  Select all that apply | *Check multiple*  (1) Hand hygiene  (2) Diarrheal diseases  (3) Respiratory infections  (4) Influenza  (5) Other Infectious diseases  (6) Nutrition  (7) Mental/social well being  (8) Neglected tropical diseases  (9) School absenteeism  (10) COVID-19  (11) Food hygiene  (12) Soil-transmitted helminth infection  (777) Other – specify  (999) Not applicable |

| **#** | **Field** | **Details** | **Entry** |
| --- | --- | --- | --- |
| 4.3 | Start date | What is the study start date? Month, Year | Text  (888) Not reported  (999) Not applicable |
| 4.4 | End date | What is the study end date? Month, Year | Text  (888) Not reported  (999) Not applicable |
| **5. Participants** | | | |
| 5.1a | Study participants- Health outcome | What group(s) of people are researchers examining the focal health outcome for in the study? Select all that apply | *Check multiple*  (1) General population  (2) Adults (Women and Men)  (3) Adults (Women only)  (4) Adults (Men only)  (5) Children (Girls and Boys)  (6) Children (Girls only)  (7) Children (Boys only)  (8) Mother-child dyads  (9) Food workers  (10) Non-food occupational workers  (777) Other – specify  (888) Not reported  (999) Not applicable |
| 5.1b | Study participants- Hand washing behavior | What group(s) of people are researchers examining hand washing behavior for in the study?  Select all that apply | *Check multiple*  (1) General population  (2) Adults (Women and Men)  (3) Adults (Women only)  (4) Adults (Men only)  (5) Children (Girls and Boys)  (6) Children (Girls only)  (7) Children (Boys only)  (8) Mother-child dyads  (9) Food workers  (10) Non-food occupational workers  (777) Other – specify  (888) Not reported  (999) Not applicable |
| **#** | **Field** | **Details** | **Entry** |
| 5.2 | Vulnerable populations | Does study concern any of the following vulnerable populations?  Select all that apply | *Check multiple*  (1) Individuals with specific illness or risk factors  (2) Specific ethnic or religious groups  (3) Persons experiencing homelessness  (4) Persons with disabilities  (5) Immigrants and migrants  (6) Refugees and displaced persons (7) Elderly  (8) Pregnant women  (777) Other - specify  (888) Not reported |
| 5.3 | Number of participants | What is the total number of participants/sample size?    If the study does not stratify participants by sex or gender, only fill in the total row. If does not stratify by adult/child, only fill in adult column. | Table  2 x 3 table for Adult/Child vs Female/Male/Total |
| 5.4 | Number of participants (mixed methods) | For mixed methods studies, how many participants are involved with each method? | Table  3x2 table for  Qual/Quant/Total |
| **6. Intervention** | | | |
| 6.1 | Theory | Did the study report using a behavior change theory? Select all that apply (model, framework) | *Select multiple*  (1) IBM-WASH  (2) RANAS  (3) Behavior Centered Design/Evo-Eco Model  (4) COM-B  (5) Theory of Planned Behavior  (6) Health Belief Model  (7) Social Ecological Model  (8) Theoretical Domains Framework  (777) Other – specify  (999) No theory reported |

| **#** | **Field** | **Details** | | **Entry** |
| --- | --- | --- | --- | --- |
| 6.2 | Theory mention | Where did the authors discuss the theory that they used? | | *Select one*  (1) Protocol  (2) Cited formative research  (3) Primary research paper  (777) Other – specify  (999) No theory reported |
| **7. Outcomes (RQ3.1)** | | | | |
| 7.1 | Physical Capability | | Were physical capability barriers or enablers presented in the study?    Definition of physical capability: physical strength, skill, stamina | (1) Yes  (0) No |
| 7.2 | Psychological Capability | | Were psychological capability barriers or enablers presented in the study?    Definition of psychological capability: knowledge/psychological strength, skills or stamina | (1) Yes  (0) No |
| 7.3 | Social opportunity | | Were social opportunity barriers or enablers presented in the study?    Definition of social opportunity: opportunities as a result of social factors, such as cultural norms and social cues | (1) Yes  (0) No |
| 7.4 | Physical opportunity | | Were physical opportunity barriers or enablers presented in the study?    Definition of physical opportunity: opportunities provided by the environment, such as time, location and resources | (1) Yes  (0) No |

| **#** | **Field** | **Details** | **Entry** |
| --- | --- | --- | --- |
| 7.5 | Automatic motivation | Were automatic motivation barriers or enablers presented in the study?    Definition of automatic motivation: automatic processes, such as our desires, impulses and inhibitions | (1) Yes  (0) No |
| 7.6 | Reflective motivation | Were reflective motivation barriers or enablers presented in the study?    Definition of reflective motivation: reflective processes, such as making plans and evaluating things that have already happened | (1) Yes  (0) No |

# MMAT: Quality-Bias assessment

| **MMAT** | | | | |
| --- | --- | --- | --- | --- |
| All articles | | | | |
| **#** | **Field** | **Details** | **Entry** | **Source** |
| S1 | Screening question 1 (for all types) | Are there clear research questions? | (0) No (1) Yes. (999) Can’t tell | [MMAT User Guide](http://mixedmethodsappraisaltoolpublic.pbworks.com/w/file/fetch/127916259/MMAT_2018_criteria-manual_2018-08-01_ENG.pdf) |
| S2 | Screening question 2 (for all types) | Do the collected data allow to address the research questions? | (0) No (1) Yes. (999) Can’t tell | [MMAT User Guide](http://mixedmethodsappraisaltoolpublic.pbworks.com/w/file/fetch/127916259/MMAT_2018_criteria-manual_2018-08-01_ENG.pdf) |
| Qualitative | | | | |
| 1.1 | Is the qualitative approach appropriate to answer the research question? | | (0) No (1) Yes. (999) Can’t tell | [MMAT User Guide](http://mixedmethodsappraisaltoolpublic.pbworks.com/w/file/fetch/127916259/MMAT_2018_criteria-manual_2018-08-01_ENG.pdf) |
| 1.2 | Are the qualitative data collection methods adequate to address the research question? | | (0) No (1) Yes. (999) Can’t tell | [MMAT User Guide](http://mixedmethodsappraisaltoolpublic.pbworks.com/w/file/fetch/127916259/MMAT_2018_criteria-manual_2018-08-01_ENG.pdf) |
| 1.3 | Are the findings adequately derived from the data? | | (0) No (1) Yes. (999) Can’t tell | [MMAT User Guide](http://mixedmethodsappraisaltoolpublic.pbworks.com/w/file/fetch/127916259/MMAT_2018_criteria-manual_2018-08-01_ENG.pdf) |
| 1.4 | Is the interpretation of results sufficiently substantiated by data? | | (0) No (1) Yes. (999) Can’t tell | [MMAT User Guide](http://mixedmethodsappraisaltoolpublic.pbworks.com/w/file/fetch/127916259/MMAT_2018_criteria-manual_2018-08-01_ENG.pdf) |
| 1.5 | Is there coherence between qualitative data sources, collection, analysis and interpretation? | | (0) No (1) Yes. (999) Can’t tell | [MMAT User Guide](http://mixedmethodsappraisaltoolpublic.pbworks.com/w/file/fetch/127916259/MMAT_2018_criteria-manual_2018-08-01_ENG.pdf) |

| **#** | **Field** | **Entry** | **Source** |
| --- | --- | --- | --- |
| Quantitative randomized controlled trials | | | |
| 2.1 | Is randomization appropriately performed? | (0) No (1) Yes. (999) Can’t tell | [MMAT User Guide](http://mixedmethodsappraisaltoolpublic.pbworks.com/w/file/fetch/127916259/MMAT_2018_criteria-manual_2018-08-01_ENG.pdf) |
| 2.2 | Are the groups comparable at baseline? | (0) No (1) Yes. (999) Can’t tell | [MMAT User Guide](http://mixedmethodsappraisaltoolpublic.pbworks.com/w/file/fetch/127916259/MMAT_2018_criteria-manual_2018-08-01_ENG.pdf) |
| 2.3 | Are there complete outcome data? | (0) No (1) Yes. (999) Can’t tell | [MMAT User Guide](http://mixedmethodsappraisaltoolpublic.pbworks.com/w/file/fetch/127916259/MMAT_2018_criteria-manual_2018-08-01_ENG.pdf) |
| 2.4 | Are outcome assessors blinded to the intervention provided? | (0) No (1) Yes. (999) Can’t tell | [MMAT User Guide](http://mixedmethodsappraisaltoolpublic.pbworks.com/w/file/fetch/127916259/MMAT_2018_criteria-manual_2018-08-01_ENG.pdf) |
| 2.5 | Did the participants adhere to the assigned intervention? | (0) No (1) Yes. (999) Can’t tell | [MMAT User Guide](http://mixedmethodsappraisaltoolpublic.pbworks.com/w/file/fetch/127916259/MMAT_2018_criteria-manual_2018-08-01_ENG.pdf) |
| Quantitative non-randomized | | | |
| 3.1 | Are the participants representative of the target population? | (0) No (1) Yes. (999) Can’t tell | [MMAT User Guide](http://mixedmethodsappraisaltoolpublic.pbworks.com/w/file/fetch/127916259/MMAT_2018_criteria-manual_2018-08-01_ENG.pdf) |
| 3.2 | Are measurements appropriate regarding both the outcome and intervention (or exposure)? | (0) No (1) Yes. (999) Can’t tell | [MMAT User Guide](http://mixedmethodsappraisaltoolpublic.pbworks.com/w/file/fetch/127916259/MMAT_2018_criteria-manual_2018-08-01_ENG.pdf) |
| 3.3 | Are there complete outcome data? | (0) No (1) Yes. (999) Can’t tell | [MMAT User Guide](http://mixedmethodsappraisaltoolpublic.pbworks.com/w/file/fetch/127916259/MMAT_2018_criteria-manual_2018-08-01_ENG.pdf) |
| 3.4 | Are the confounders accounted for in the design and analysis? | (0) No (1) Yes. (999) Can’t tell | [MMAT User Guide](http://mixedmethodsappraisaltoolpublic.pbworks.com/w/file/fetch/127916259/MMAT_2018_criteria-manual_2018-08-01_ENG.pdf) |
| 3.5 | During the study period, is the intervention administered (or exposure occurred) as intended? | (0) No (1) Yes. (999) Can’t tell | [MMAT User Guide](http://mixedmethodsappraisaltoolpublic.pbworks.com/w/file/fetch/127916259/MMAT_2018_criteria-manual_2018-08-01_ENG.pdf) |
| Quantitative descriptive | | | |
| 4.1 | Is the sampling strategy relevant to address the research question? | (0) No (1) Yes. (999) Can’t tell | [MMAT User Guide](http://mixedmethodsappraisaltoolpublic.pbworks.com/w/file/fetch/127916259/MMAT_2018_criteria-manual_2018-08-01_ENG.pdf) |
| 4.2 | Is the sample representative of the target population? | (0) No (1) Yes. (999) Can’t tell | [MMAT User Guide](http://mixedmethodsappraisaltoolpublic.pbworks.com/w/file/fetch/127916259/MMAT_2018_criteria-manual_2018-08-01_ENG.pdf) |
| 4.3 | Are the measurements appropriate? | (0) No (1) Yes. (999) Can’t tell | [MMAT User Guide](http://mixedmethodsappraisaltoolpublic.pbworks.com/w/file/fetch/127916259/MMAT_2018_criteria-manual_2018-08-01_ENG.pdf) |
| 4.4 | Is the risk of nonresponse bias low? | (0) No (1) Yes. (999) Can’t tell | [MMAT User Guide](http://mixedmethodsappraisaltoolpublic.pbworks.com/w/file/fetch/127916259/MMAT_2018_criteria-manual_2018-08-01_ENG.pdf) |
| 4.5 | Is the statistical analysis appropriate to answer the research question? | (0) No (1) Yes. (999) Can’t tell | [MMAT User Guide](http://mixedmethodsappraisaltoolpublic.pbworks.com/w/file/fetch/127916259/MMAT_2018_criteria-manual_2018-08-01_ENG.pdf) |
| Mixed methods | | | |
| 5.1 | Is there an adequate rationale for using a mixed methods design to address the research question? | (0) No (1) Yes. (999) Can’t tell | [MMAT User Guide](http://mixedmethodsappraisaltoolpublic.pbworks.com/w/file/fetch/127916259/MMAT_2018_criteria-manual_2018-08-01_ENG.pdf) |
| 5.2 | Are the different components of the study effectively integrated to answer the research question? | (0) No (1) Yes. (999) Can’t tell | [MMAT User Guide](http://mixedmethodsappraisaltoolpublic.pbworks.com/w/file/fetch/127916259/MMAT_2018_criteria-manual_2018-08-01_ENG.pdf) |
| 5.3 | Are the outputs of the integration of qualitative and quantitative components adequately interpreted? | (0) No (1) Yes. (999) Can’t tell | [MMAT User Guide](http://mixedmethodsappraisaltoolpublic.pbworks.com/w/file/fetch/127916259/MMAT_2018_criteria-manual_2018-08-01_ENG.pdf) |
| 5.4 | Are divergences and inconsistencies between quantitative and qualitative results adequately addressed? | (0) No (1) Yes. (999) Can’t tell | [MMAT User Guide](http://mixedmethodsappraisaltoolpublic.pbworks.com/w/file/fetch/127916259/MMAT_2018_criteria-manual_2018-08-01_ENG.pdf) |
| 5.5 | Do the different components of the study adhere to the quality criteria of each tradition of the methods involved? | (0) No (1) Yes. (999) Can’t tell | [MMAT User Guide](http://mixedmethodsappraisaltoolpublic.pbworks.com/w/file/fetch/127916259/MMAT_2018_criteria-manual_2018-08-01_ENG.pdf) |
