# Supplementary material for: Behavioural factors influencing hand hygiene practices across domestic, institutional and public community settings: a systematic review and qualitative meta-synthesis
Source: BMJ Glob Health. 2025 Sep 16;10(Suppl 7):e018927. doi: 10.1136/bmjgh-2025-018927 (PMC12443170; doi:10.1136/bmjgh-2025-018927)
Supplement: online supplemental file 11 [file bmjgh-10-Suppl_7-s011.docx]

**Behavioural factors influencing hand hygiene practices across domestic, institutional, and public community settings: A systematic review and qualitative meta-synthesis**

Bethany A. Caruso^1^, Jedidiah S. Snyder^2^, Lilly A. O’Brien^2^, Erin LaFon^2^ , Kennedy Files^2^, Dewan Muhammad Shoaib^1^, Sridevi K. Prasad^1^ , Hannah Rogers^3^ , Oliver Cumming^4,5^, Joanna Esteves Mills^5^, Bruce Gordon ^5^, Marlene K. Wolfe^2*^, Matthew C. Freeman^2*^

1 Hubert Department of Global Health, Rollins School of Public Health, Emory University, Atlanta, GA, USA;

2 Gangarosa Department of Environmental Health, Rollins School of Public Health, Emory University, Atlanta, GA, USA;

3 Woodruff Health Sciences Center Library, Emory University, Atlanta, GA, USA;

4 Department of Disease Control, London School of Hygiene and Tropical Medicine, London, UK;

5 Water, Sanitation, Hygiene and Health Unit, World Health Organization, Geneva, Switzerland.

Corresponding author: Bethany A. Caruso; bcaruso@emory.edu

Emory University, Rollins School of Public Health, 1518 Clifton Rd, Atlanta, GA 30322

*Contributed equally.

**GRADE-CERQual Assessment of confidence in evidence for thematic findings, by COM-B theme and setting**

| **Summary of review finding** | **Setting** | **Barrier/ Enabler** | **Studies contributing to finding** | **Mean Theme**  **MMAT Score** | **Methodological limitations^1,2^ (Informed by  MMAT)**^3^ | **Coherence^1,2^**  Definition:  An assessment of how clear and cogent the fit is between the data from the primary studies and a review finding that synthesises that data. By ‘cogent’, we mean well supported or compelling. | **Adequacy^,1,2^**  Definition:  An overall determination of the degree of richness and quantity of data supporting a review finding. | **Relevance^,1,2^**  Definition:  The extent to which the body of evidence from the primary studies supporting a review finding is applicable to the context (perspective or population, phenomenon of interest, setting) specified in the review question. | **Overall GRADE-CERQual confidence assessment^4^** | **Explanation of GRADE-CERQual assessment** |
| --- | --- | --- | --- | --- | --- | --- | --- | --- | --- | --- |
| **CAPABILITY** |  |  |  |  |  |  |  |  |  |  |
| **Physical Capability** | |  |  |  | |  |  |  |  |  |
| **Ease/ difficulty of washing hands** | **Domestic** | Barrier | Mitchell 2021 | 5 | No concerns | Serious concerns | Serious concerns | Serious concerns | Very Low | Very small number of studies limiting coherence, adequacy, and geographical/ population relevance. |
|  |  | Enabler | x | x | x | x | x | x | x | x |
|  | **Institutional** | Barrier | x | x | x | x | x | x | x | x |
|  |  | Enabler | x | x | x | x | x | x | x | x |
|  | **Public** | Barrier | Nizame 2019 | 5 | No concerns | Serious concerns | Serious concerns | Serious concerns | Very Low | Very small number of studies limiting coherence, adequacy, and geographical/ population relevance. |
|  |  | Enabler | x | x | x | x | x | x | x | x |
| **Ease/ difficulty of collecting water** | **Domestic** | Barrier | Akter 2014  Mohamed 2022 | 4 | Minor concerns | Serious concerns | Serious concerns | Serious concerns | Very Low | Very small number of studies limiting coherence, adequacy and geographical/ population relevance. |
|  |  | Enabler | x | x | x | x | x | x | x | x |
|  | **Institutional** | Barrier | x | x | x | x | x | x | x | x |
|  |  | Enabler | x | x | x | x | x | x | x | x |
|  | **Public** | Barrier | x | x | x | x | x | x | x | x |
|  |  | Enabler | x | x | x | x | x | x | x | x |

| **Summary of review finding** | **Setting** | **Barrier/ Enabler** | **Studies contributing to finding** | **Mean Theme**  **MMAT Score** | **Methodological limitations^1,2^ (Informed by  MMAT)**^3^ | **Coherence ^1,2^** | **Adequacy^, 1,2^** | **Relevance^, 1,2^** | **Overall GRADE-CERQual confidence assessment^4^** | **Explanation of GRADE-CERQual assessment** |
| --- | --- | --- | --- | --- | --- | --- | --- | --- | --- | --- |
| **Psychological Capability** | | | | | | | | | | |
| **Action Knowledge** | **Domestic** | Barrier | Akter 2014  Biran 2005  Didier 2021  Hoque 2023  Langford 2013  Mitchell 2021 | 5 | No concerns | Moderate concerns | Moderate concerns | Serious concerns | Moderate | Number of studies limiting coherence, adequacy, and geographical/population relevance (half in Asia, none in Africa; limited urban). |
|  |  | Enabler | Akter 2014  Bauza 2021 Ogutu 2022  Rahman 2017  Sedekia 2022  Zangana 2020 | 5 | No concerns | Moderate concerns | Moderate concerns | Serious concerns | Moderate | Number of studies limiting coherence, adequacy, and geographical/population relevance (most Africa/Asia, none in HICs; limited urban). |
|  | **Institutional** | Barrier | Arendt 2015  Kumar 2018  Schmidt 2009 | 5 | No concerns | Moderate concerns | Serious concerns | Serious concerns | Very Low | Very small number of studies limiting coherence, adequacy, and geographical/ population relevance (most in HICs; most in school settings). |
|  |  | Enabler | Babalobi 2013  La Con 2017  Green 2005  Mbakaya 2019  Okello 2019  Sebong 2021 | 5 | No concerns | Moderate concerns | Serious concerns | Serious concerns | Moderate | Number of studies limiting coherence, adequacy, and geographical/population relevance (half in Africa, many regions missing; most in education settings). |
|  | **Public** | Barrier | x | x | x | x | x | x | x | x |
|  |  | Enabler | x | x | x | x | x | x | x | x |

| **Summary of review finding** | **Setting** | **Barrier/ Enabler** | **Studies contributing to finding** | **Mean Theme**  **MMAT Score** | **Methodological limitations^1,2^ (Informed by  MMAT)**^3^ | **Coherence ^1,2^** | **Adequacy^, 1,2^** | **Relevance^, 1,2^** | **Overall GRADE-CERQual confidence assessment^4^** | **Explanation of GRADE-CERQual assessment** |
| --- | --- | --- | --- | --- | --- | --- | --- | --- | --- | --- |
| **OPPORTUNITY** | | | | | | | | | | |
| **Physical Opportunity** | | | | | | | | | | |
| **Soap/ Sanitizer availability** | **Domestic** | Barrier | Afolabi 2022  Biswas 2017  Didier 2021  Greenwell 2013  Kalumbi 2020  Lanfer 2021  Langford 2013  Lohiniva 2007  Mohamed 2022  Sagan 2019  Parveen 2018  Sultana 2018  White 2022 B | 4.7 | Very minor concerns | No concerns | No concerns | Very minor concerns | High | Overall, adequate number of studies supporting theme across multiple settings. Some limitation in geographic relevance (HIC limited; most Africa, Asia). |
|  |  | Enabler | Biran 2005  Biswas 2017  Kalam 2021  Mohamed 2022  Sultana 2018  Zangana 2020 | 4.7 | Very minor concerns | No concerns | Very minor concerns | Moderate concerns | Moderate | Limitation in geographic relevance (No HIC; most Africa, Asia). |
|  | **Institutional** | Barrier | Al-Naggar 2013  Devkota 2020  Kumar 2018  Melaku 2023  Okello 2019  Sebong 2021  Steenkamp 2022 | 4.6 | Very minor concerns | No concerns | Very minor concerns | Moderate concerns | Moderate | Limitation in geographic relevance (No HIC; all education settings). |
|  |  | Enabler | Jackson 2021  Mbakaya 2019 | 5 | No concerns | Moderate concerns | Serious concerns | Serious concerns | Very Low | Very small number of studies limiting coherence, adequacy, and geographical/ population relevance (1 US, 1 Africa, all schools). |

| **Summary of review finding** | **Setting** | **Barrier/ Enabler** | **Studies contributing to finding** | | **Mean Theme**  **MMAT Score** | | **Methodological limitations^1,2^ (Informed by  MMAT)**^3^ | **Coherence ^1,2^** | **Adequacy^, 1,2^** | **Relevance^, 1,2^** | **Overall GRADE-CERQual confidence assessment^4^** | **Explanation of GRADE-CERQual assessment** |
| --- | --- | --- | --- | --- | --- | --- | --- | --- | --- | --- | --- | --- |
| **Soap/ Sanitizer availability (continued)** | **Public** | Barrier | Babalobi 2013  Biran 2012  Blum 2019  Claude 2020  Thorseth 2021  Wu 2019 | 5 | | No concerns | | Moderate concerns | Minor concerns | Serious concerns | Moderate | Number of studies limiting adequacy and geographical/population relevance (all Africa; half IDP). |
|  |  | Enabler | Neetu 2013  Thorseth 2021 | 5 | | No concerns | | Moderate concerns | Serious concerns | Serious concerns | Very Low | Very limited number of studies limiting adequacy and geographical/population relevance (no HICs; one market,1 IDP). |
| **Soap cost** | **Domestic** | Barrier | Aberese-Ako 2023  Affleck 2012  Afolabi 2022  Akter 2022  Azam 2022  Bauza 2021  Biran 2005  Chidziwisano 2019  Biswas 2017  Grant 2023  Hoque 2023  Lanfer 2021  Langford 2013  Mohamed 2022  Nizame 2016  Ogutu 2022  Scott 2007  Parveen 2018  White 2022 A  Yallew 2012 | 4.9 | | Very minor concerns | | No concerns | No concerns | Minor concerns | High | Overall, adequate number of studies supporting theme across multiple settings. Limitation in geographic relevance (No HIC; most Africa, Asia). |

| **Summary of review finding** | **Setting** | **Barrier/ Enabler** | **Studies contributing to finding** | **Mean Theme**  **MMAT Score** | **Methodological limitations^1,2^ (Informed by  MMAT)**^3^ | **Coherence ^1,2^** | **Adequacy^, 1,2^** | **Relevance^, 1,2^** | | **Overall GRADE-CERQual confidence assessment^4^** | | **Explanation of GRADE-CERQual assessment** |
| --- | --- | --- | --- | --- | --- | --- | --- | --- | --- | --- | --- | --- |
| **Soap cost**  **(continued)** | **Domestic (continued)** | Enabler | Biswas 2017  Ogutu 2022  Ashraf 2017 | 5 | No concerns | Minor concerns | Serious concerns | | Serious concerns | | Low | Number of studies limiting adequacy and geographical/population relevance (No HICs; all rural, per-urban). |
|  | **Institutional** | Barrier | Devkota 2020 | 2 | Serious concerns | Moderate concerns | Serious concerns | | Serious concerns | | Very Low | Number of studies limiting adequacy and geographical/population relevance (only Nepal, only school). |
|  |  | Enabler | x | x | x | x | x | | x | | x | x |
|  | **Public** | Barrier | Blum 2019  Nizame 2019  Thorseth 2021 | 5 | No concerns | Minor concerns | Serious concerns | | Serious concerns | | Low | Number of studies limiting adequacy and geographical/population relevance (no HICs; 1 market; IDPs). |
|  |  | Enabler | x | x | x | x | x | | x | | x | x |
| **Water availability** | **Domestic** | Barrier | Akter 2014  Atuyambe 2011  Biran 2005  Demberere 2016  Didier 2021  Greenwell 2013  Lanfer 2021  Sagan 2019 | 5 | No concerns | No concerns | Very minor concerns | | Minor concerns | | High | Overall, adequate number of studies supporting theme across multiple settings. Limitation in geographic relevance (most Africa, Asia). |
|  |  | Enabler | Dearden 2002  Kalam 2021  Ashraf 2017 | 5 | No concerns | Moderate concerns | Serious concerns | | Serious concerns | | Very Low | Limitation in geographic relevance (No HIC; all Asia). |
|  | **Institutional** | Barrier | Xuan 2013  Melaku 2023  Okello 2019 | 5 | No concerns | Minor concerns | Serious concerns | | Serious concerns | | Low | Limitation in geographic relevance (No HIC; all schools). |
|  |  | Enabler | Okello 2019  Steenkamp 2022 | 5 | No concerns | Moderate concerns | Serious concerns | | Serious concerns | | Very Low | Limitation in geographic relevance (all Africa; all schools). |

| **Summary of review finding** | **Setting** | **Barrier/ Enabler** | **Studies contributing to finding** | **Mean Theme**  **MMAT Score** | **Methodological limitations^1,2^ (Informed by  MMAT)**^3^ | **Coherence ^1,2^** | **Adequacy^, 1,2^** | **Relevance^, 1,2^** | **Overall GRADE-CERQual confidence assessment^4^** | **Explanation of GRADE-CERQual assessment** |
| --- | --- | --- | --- | --- | --- | --- | --- | --- | --- | --- |
| **Water availability**  **(continued)** | **Public** | Barrier | Babalobi 2013  Claude 2020 | 5 | No concerns | Moderate concerns | Serious concerns | Serious concerns | Very Low | Limitation in geographic relevance (all Africa; 1 market, 2 IDP). |
|  |  | Enabler | x | x | x | x | x | x | x | x |
| **Water supply quantity** | **Domestic** | Barrier | Aberese-Ako 2023  Akter 2014  Atuyambe 2011  Langford 2013 Lohiniva 2007  Scott 2007  White 2022 B | 4.9 | Very minor concerns | No concerns | Minor concerns | Moderate concerns | High | Limitation in geographic relevance (No HIC; majority Africa). |
|  |  | Enabler | x | x | x | x | x | x | x | x |
|  | **Institutional** | Barrier | Al-Naggar 2013  Melaku 2023 | 5 | No concerns | Moderate concerns | Serious concerns | Serious concerns | Very Low | Limitation in geographic relevance (No HIC; Education settings only). |
|  |  | Enabler | x | x | x | x | x | x | x | x |
|  | **Public** | Barrier | x | x | x | x | x | x | x | x |
|  |  | Enabler | x | x | x | x | x | x | x | x |
| **Water distance** | **Domestic** | Barrier | Dearden 2002  Demberere 2016  Grant 2023  Mohamed 2022  Parveen 2018 | 4.6 | Very minor concerns | No concerns | Minor concerns | Moderate concerns | Moderate | Limitation in geographic relevance (No HIC; Africa/Asia only). |
|  |  | Enabler | Dearden 2002  Biswas 2017 | 5 | No concerns | Moderate concerns | Serious concerns | Serious concerns | Very Low | Limitation in geographic relevance (No HIC; Asia only; rural only). |
|  | **Institutional** | Barrier | Al-Naggar 2013 | 5 | No concerns | Moderate concerns | Serious concerns | Serious concerns | Very Low | Limitation in geographic relevance (No HIC; Asia only; University only). |
|  |  | Enabler | x | x | x | x | x | x | x | x |
|  | **Public** | Barrier | x | x | x | x | x | x | x | x |
|  |  | Enabler | x | x | x | x | x | x | x | x |

| **Summary of review finding** | **Setting** | **Barrier/ Enabler** | **Studies contributing to finding** | **Mean Theme**  **MMAT Score** | **Methodological limitations^1,2^ (Informed by  MMAT)**^3^ | **Coherence ^1,2^** | **Adequacy^, 1,2^** | **Relevance^, 1,2^** | **Overall GRADE-CERQual confidence assessment^4^** | **Explanation of GRADE-CERQual assessment** |
| --- | --- | --- | --- | --- | --- | --- | --- | --- | --- | --- |
| **Water cost** | **Domestic** | Barrier | Mohamed 2022  Yallew 2012 | 4 | Minor concerns | Moderate concerns | Serious concerns | Serious concerns | Very Low | Study quality concerning; number of studies limiting adequacy and geographic relevance (only Africa). |
|  |  | Enabler | x | x | x | x | x | x | x | x |
|  | **Institutional** | Barrier | x | x | x | x | x | x | x | x |
|  |  | Enabler | x | x | x | x | x | x | x | x |
|  | **Public** | Barrier | x | x | x | x | x | x | x | x |
|  |  | Enabler | x | x | x | x | x | x | x | x |
| **Household infrastructure** | **Domestic** | Barrier | Atuyambe 2011  Biswas 2017  Didier 2021  Hoque 2023  Lohiniva 2007 | 5 | No concerns | Minor concerns | Minor concerns | Minor concerns | High | Overall, adequate number of studies supporting theme across multiple settings. Limitation in geographic relevance (mostly rural). |
|  |  | Enabler | Biswas 2017 | 5 | No concerns | Moderate concerns | Serious concerns | Serious concerns | Very Low | Limited number of studies limiting adequacy and geographical/population relevance. |
|  | **Institutional** | Barrier | x | x | x | x | x | x | x | x |
|  |  | Enabler | x | x | x | x | x | x | x | x |
|  | **Public** | Barrier | x | x | x | x | x | x | x | x |
|  |  | Enabler | x | x | x | x | x | x | x | x |

| **Summary of review finding** | **Setting** | **Barrier/ Enabler** | **Studies contributing to finding** | **Mean Theme**  **MMAT Score** | **Methodological limitations^1,2^ (Informed by  MMAT)**^3^ | **Coherence ^1,2^** | **Adequacy^, 1,2^** | **Relevance^, 1,2^** | **Overall GRADE-CERQual confidence assessment^4^** | **Explanation of GRADE-CERQual assessment** |
| --- | --- | --- | --- | --- | --- | --- | --- | --- | --- | --- |
| **Community infrastructure** | **Domestic** | Barrier | x | x | x | x | x | x | x | x |
|  |  | Enabler | x | x | x | x | x | x | x | x |
|  | **Institutional** | Barrier | La Con 2017  Green 2005  Kumar 2018  Scott 2007  Sebong 2021  Steiner-Asiedu 2011 | 5 | No concerns | Minor concerns | Minor concerns | Minor concerns | High | Overall, adequate number of studies supporting theme across multiple settings. Limitation in geographic relevance (mostly LMICs, mostly schools). |
|  |  | Enabler | Jackson 2021 | 5 | No concerns | Moderate concerns | Serious concerns | Serious concerns | Very Low | Number of studies limiting adequacy and geographical/population relevance (USA only, athletic facility only.) |
|  | **Public** | Barrier | Aberese-Ako 2023  Scott 2007  Yardley 2011 | 5 | No concerns | Minor concerns | Serious concerns | Serious concerns | Low | Number of studies limiting adequacy and geographical/population relevance. |
|  |  | Enabler | x | x | x | x | x | x | x | x |
| **Hand hygiene station location** | **Domestic** | Barrier | Akter 2022  Chidziwisano 2019  Hoque 2023 Nizame 2013  Ashraf 2017  Steiner-Asiedu 2011 | 5 | No concerns | Minor concerns | Minor concerns | Minor concerns | High | Overall, adequate number of studies supporting theme across multiple settings. Limitation in geographic relevance (mostly Asia, all rural). |
|  |  | Enabler | Biswas 2017  Ashraf 2017  Tibbels 2022 | 5 | No concerns | Minor concerns | Serious concerns | Serious concerns | Low | Limitation in geographic relevance (LMIC only). |
|  | **Institutional** | Barrier | Al-Naggar 2013  La Con 2017  Green 2005  Jackson 2021 | 5 | No concerns | Minor concerns | Moderate concerns | Moderate concerns | Moderate | Limitation in geographic relevance (mostly education settings). |
|  |  | Enabler | Jackson 2021  Okello 2019  Pragle 2007 | 5 | No concerns | Minor concerns | Serious concerns | Serious concerns | Low | Limitation in geographic relevance (LMIC and HIC but limited number; schools and workplace but limited). |

| **Summary of review finding** | **Setting** | **Barrier/ Enabler** | **Studies contributing to finding** | **Mean Theme**  **MMAT Score** | **Methodological limitations^1,2^ (Informed by  MMAT)**^3^ | **Coherence ^1,2^** | **Adequacy^, 1,2^** | **Relevance^, 1,2^** | **Overall GRADE-CERQual confidence assessment^4^** | **Explanation of GRADE-CERQual assessment** |
| --- | --- | --- | --- | --- | --- | --- | --- | --- | --- | --- |
| **Hand hygiene station location**  **(continued)** | **Public** | Barrier | Watson 2020 | 5 | No concerns | Minor concerns | Serious concerns | Serious concerns | Very Low | Number of studies limiting adequacy and geographical/population relevance (IDP only) |
|  |  | Enabler | Neetu 2013 | 5 | No concerns | Minor concerns | Serious concerns | Serious concerns | Very Low | Number of studies limiting adequacy and geographical/population relevance (market only). |
| **Hand hygiene station design** | **Domestic** | Barrier | Hoque 2023  Mohamed 2022  White 2022 B  Zangana 2020 | 4.3 | Minor concerns | Minor concerns | Moderate concerns | Moderate concerns | Moderate | Limitation in geographic relevance (mostly Africa; LMICs only). |
|  |  | Enabler | White 2022 A  White 2022 B | 4.5 | Very minor concerns | Moderate concerns | Serious concerns | Serious concerns | Very Low | Number of studies limiting coherence, adequacy and geographical/population relevance. |
|  | **Institutional** | Barrier | x | x | x | x | x | x | x | x |
|  |  | Enabler | x | x | x | x | x | x | x | x |
|  | **Public** | Barrier | Mohamed 2022 | 3 | Moderate concerns | Moderate concerns | Serious concerns | Serious concerns | Very Low | Low quality and number of studies limiting coherence, adequacy and geographical/ population relevance. |
|  |  | Enabler | x | x | x | x | x | x | x | x |
| **State of hand hygiene equipment** | **Domestic** | Barrier | White 2020 B | 4 | Minor concerns | Moderate concerns | Serious concerns | Serious concerns | Very low | Limited number of studies limiting coherence, adequacy and geographical/ population relevance. |
|  |  | Enabler | x | x | x | x | x | x | x | x |

| **Summary of review finding** | **Setting** | **Barrier/ Enabler** | **Studies contributing to finding** | **Mean Theme**  **MMAT Score** | **Methodological limitations^1,2^ (Informed by  MMAT)**^3^ | **Coherence ^1,2^** | **Adequacy^, 1,2^** | **Relevance^, 1,2^** | **Overall GRADE-CERQual confidence assessment^4^** | **Explanation of GRADE-CERQual assessment** |
| --- | --- | --- | --- | --- | --- | --- | --- | --- | --- | --- |
| **State of hand hygiene equipment**  **(Continued)** | **Institutional** | Barrier | Melaku 2023 | 5 | No concerns | Moderate concerns | Serious concerns | Serious concerns | Very low | Limited number of studies limiting coherence, adequacy and geographical/ population relevance. |
|  |  | Enabler | Melaku 2023 | 5 | No concerns | Moderate concerns | Serious concerns | Serious concerns | Very low | Limited number of studies limiting coherence, adequacy and geographical/ population relevance. |
|  | **Public** | Barrier | Blum 2019 | 5 | No concerns | Moderate concerns | Serious concerns | Serious concerns | Very low | Limited number of studies limiting coherence, adequacy and geographical/ population relevance. |
|  |  | Enabler | x | x | x | x | x | x | x | x |
| **Cleanliness of hand washing environment** | **Domestic** | Barrier | Zangana 2020 | 5 | No concerns | Moderate concern | Serious concerns | Serious concerns | Very low | Limited number of studies limiting coherence, adequacy and geographical/ population relevance. |
|  |  | Enabler | x | x | x | x | x | x | x | x |
|  | **Institutional** | Barrier | Kumar 2018 | 5 | No concerns | Moderate concern | Serious concerns | Serious concerns | Very low | Limited number of studies limiting coherence, adequacy and geographical/ population relevance. |
|  |  | Enabler | x | x | x | x | x | x | x | x |
|  | **Public** | Barrier | Wu 2019 | 5 | No concerns | Moderate concern | Serious concerns | Serious concerns | Very low | Limited number of studies limiting coherence, adequacy and geographical/ population relevance. |
|  |  | Enabler | x | x | x | x | x | x | x | x |

| **Summary of review finding** | **Setting** | **Barrier/ Enabler** | **Studies contributing to finding** | **Mean Theme**  **MMAT Score** | **Methodological limitations^1,2^ (Informed by  MMAT)**^3^ | **Coherence ^1,2^** | **Adequacy^, 1,2^** | **Relevance^, 1,2^** | **Overall GRADE-CERQual confidence assessment^4^** | **Explanation of GRADE-CERQual assessment** |
| --- | --- | --- | --- | --- | --- | --- | --- | --- | --- | --- |
| **Lighting of hand washing area** | **Domestic** | Barrier | x | x | x | x | x | x | x | x |
|  |  | Enabler | x | x | x | x | x | x | x | x |
|  | **Institutional** | Barrier | x | x | x | x | x | x | x | x |
|  |  | Enabler | x | x | x | x | x | x | x | x |
|  | **Public** | Barrier | Watson 2020 | 5 | No concerns | Moderate concern | Serious concerns | Serious concerns | Very low | Limited number of studies limiting coherence, adequacy and geographical/ population relevance. |
|  |  | Enabler | x | x | x | x | x | x | x | x |
| **Damage of resources** | **Domestic** | Barrier | White 2022 A | 5 | No concerns | Moderate concern | Serious concerns | Serious concerns | Very low | Limited number of studies limiting coherence, adequacy and geographical/ population relevance. |
|  |  | Enabler | x | x | x | x | x | x | x | x |
|  | **Institutional** | Barrier | La Con 2017  Melaku 2023  Pragle 2007 | 5 | No concerns | Moderate concern | Serious concerns | Serious concerns | Low | Limited number of studies limiting coherence, adequacy and geographical/ population relevance. |
|  |  | Enabler | x | x | x | x | x | x | x | x |
|  | **Public** | Barrier | x | x | x | x | x | x | x | x |
|  |  | Enabler | x | x | x | x | x | x | x | x |
| **Theft of resources** | **Domestic** | Barrier | Akter 2014  Biswas 2017  Ntakirutimana 2021  Sultana 2018 | 5 | No concerns | Moderate concern | Moderate concern | Serious concerns | Low | Limited number of studies limiting coherence, adequacy and geographical/ population relevance (3 of 4 in Bangladesh). |
|  |  | Enabler | x | x | x | x | x | x | x | x |
|  | **Institutional** | Barrier | x | x | x | x | x | x | x | x |
|  |  | Enabler | x | x | x | x | x | x | x | x |
|  | **Public** | Barrier | x | x | x | x | x | x | x | x |
|  |  | Enabler | x | x | x | x | x | x | x | x |

| **Summary of review finding** | **Setting** | **Barrier/ Enabler** | **Studies contributing to finding** | **Mean Theme**  **MMAT Score** | **Methodological limitations^1,2^ (Informed by  MMAT)**^3^ | **Coherence ^1,2^** | **Adequacy^, 1,2^** | **Relevance^, 1,2^** | **Overall GRADE-CERQual confidence assessment^4^** | **Explanation of GRADE-CERQual assessment** |
| --- | --- | --- | --- | --- | --- | --- | --- | --- | --- | --- |
| **Resources available for purchase** | **Domestic** | Barrier | Yeasmin 2021  Torres-Slimming 2019 | 4 | Minor concerns | Moderate concern | Serious concerns | Serious concerns | Very low | Limited number of studies limiting coherence, adequacy and geographical/ population relevance |
|  |  | Enabler | x | x | x | x | x | x | x | x |
|  | **Institutional** | Barrier | x | x | x | x | x | x | x | x |
|  |  | Enabler | x | x | x | x | x | x | x | x |
|  | **Public** | Barrier | Thorseth 2021 | 5 | No concerns | Moderate concern | Serious concerns | Serious concerns | Very low | Limited number of studies limiting coherence, adequacy and geographical/ population relevance |
|  |  | Enabler | x | x | x | x | x | x | x | x |
| **Social Opportunity** |  | |  |  | |  |  |  |  |  |
| **Role model for behavior** | **Domestic** | Barrier | Lanfer 2021 | 5 | No concerns | Moderate concerns | Serious concerns | Serious concerns | Very low | Number of studies limiting coherence, adequacy and geographical/population relevance |
|  |  | Enabler | Yeasmin 2021  Langford 2013  Sagan 2019  Ashraf 2017 | 4.3 | Minor concerns | Moderate concerns | Moderate concerns | Serious concerns | Low | Number of studies limiting coherence, adequacy and geographical/population relevance (all Bangladesh, Nepal, Pakistan) |
|  | **Institutional** | Barrier | x | x | x | x | x | x | x | x |
|  |  | Enabler | x | x | x | x | x | x | x | x |
|  | **Public** | Barrier | x | x | x | x | x | x | x | x |
|  |  | Enabler | x | x | x | x | x | x | x | x |

| **Summary of review finding** | **Setting** | **Barrier/ Enabler** | **Studies contributing to finding** | **Mean Theme**  **MMAT Score** | **Methodological limitations^1,2^ (Informed by  MMAT)**^3^ | **Coherence ^1,2^** | **Adequacy^, 1,2^** | **Relevance^, 1,2^** | **Overall GRADE-CERQual confidence assessment^4^** | **Explanation of GRADE-CERQual assessment** |
| --- | --- | --- | --- | --- | --- | --- | --- | --- | --- | --- |
| **Social Pressure** | **Domestic** | Barrier | Langford 2013  Parveen 2018 | 5 | No concerns | Moderate concerns | Serious concerns | Serious concerns | Very Low | Number of studies limiting coherence, adequacy, and geographical/population relevance |
|  |  | Enabler | Afolabi 2022  Harrison 2019  Langford 2013  Thaivalappil 2022  Tibbels 2022 | 5 | No concerns | Moderate concerns | Minor concerns | Moderate concerns | Moderate | Number of studies limiting coherence, adequacy, and geographical/population relevance |
|  | **Institutional** | Barrier | x | x | x | x | x | x | x | x |
|  |  | Enabler | Green 2005  Mbakaya 2019  Pragle 2007  Schmidt 2009 | 5 | No concerns | Moderate concerns | Minor concerns | Moderate concerns | Moderate | Number of studies limiting coherence, adequacy, and geographical/population relevance (3 in HICs, 2 in Africa; School / workplace mix) |
|  | **Public** | Barrier | x | x | x | x | x | x | x | x |
|  |  | Enabler | Blum 2019 | 5 | No concerns | Moderate concerns | Serious concerns | Serious concerns | Very low | Number of studies limiting adequacy and geographical/population relevance. |
| **Visits by Authorities** | **Domestic** | Barrier | x | x | x | x | x | x | x | x |
|  |  | Enabler | Ward 2022 | 5 | No concerns | Serious concerns | Serious concerns | Serious concerns | Very low | Number of studies limiting adequacy and geographical/population relevance Urban Nigeria only; COVID-related). |
|  | **Institutional** | Barrier | x | x | x | x | x | x | x | x |
|  |  | Enabler | Sebong 2021 | 5 | No concerns | Serious concerns | Serious concerns | Serious concerns | Very low | Number of studies limiting adequacy and geographical/population relevance (university in Asia only; COVID-related). |
|  | **Public** | Barrier | x | x | x | x | x | x | x | x |
|  |  | Enabler | x | x | x | x | x | x | x | x |

| **Summary of review finding** | **Setting** | **Barrier/ Enabler** | **Studies contributing to finding** | **Mean Theme**  **MMAT Score** | **Methodological limitations^1,2^ (Informed by  MMAT)**^3^ | **Coherence ^1,2^** | **Adequacy^, 1,2^** | **Relevance^, 1,2^** | **Overall GRADE-CERQual confidence assessment^4^** | **Explanation of GRADE-CERQual assessment** |
| --- | --- | --- | --- | --- | --- | --- | --- | --- | --- | --- |
| **Social stigmatization/ status** | **Domestic** | Barrier | Azam 2022  Parveen 2018  White 2022 B  Yardley 2011 | 4.8 | Very minor concerns | Moderate concerns | Moderate concerns | Moderate concerns | Low | Number of studies limiting adequacy and geographical/population relevance. |
|  |  | Enabler | Langford 2013  Sagan 2019  Scott 2007  Tibbels 2022  White 2022 A  White 2022 B  Zangana 2020 | 4.9 | Very minor concerns | Minor concerns | Very minor concerns | Moderate concerns | Moderate | Number of studies limiting adequacy and geographical/population relevance (all LMICs). |
|  | **Institutional** | Barrier | Kumar 2018 | 5 | No concerns | Moderate concerns | Serious concerns | Serious concerns | Very low | Number of studies limiting adequacy and geographical/population relevance. |
|  |  | Enabler | Green 2005  Jackson 2021 | 5 | No concerns | Moderate concerns | Serious concerns | Serious concerns | Very low | Number of studies limiting adequacy and geographical/population relevance. |
|  | **Public** | Barrier | x | x | x | x | x | x | x | x |
|  |  | Enabler | Blum 2019 Watson 2020 | 5 | No concerns | Moderate concerns | Serious concerns | Serious concerns | Very low | Number of studies limiting adequacy and geographical/population relevance. |
| **Informational support** | **Domestic** | Barrier | Mohamed 2022 | 3 | Moderate concerns | Moderate concerns | Serious concerns | Serious concerns | Very low | Number of studies limiting adequacy and geographical/population relevance. |
|  |  | Enabler | Afolabi 2022  Torres-Slimming 2019 | 5 | No concerns | Moderate concerns | Serious concerns | Serious concerns | Very low | Number of studies limiting adequacy and geographical/population relevance. |
|  | **Institutional** | Barrier | x | x | x | x | x | x | x | x |
|  |  | Enabler | x | x | x | x | x | x | x | x |
|  | **Public** | Barrier | x | x | x | x | x | x | x | x |
|  |  | Enabler | x | x | x | x | x | x | x | x |

| **Summary of review finding** | **Setting** | **Barrier/ Enabler** | **Studies contributing to finding** | **Mean Theme**  **MMAT Score** | **Methodological limitations^1,2^ (Informed by  MMAT)**^3^ | **Coherence ^1,2^** | **Adequacy^, 1,2^** | **Relevance^, 1,2^** | **Overall GRADE-CERQual confidence assessment^4^** | **Explanation of GRADE-CERQual assessment** |
| --- | --- | --- | --- | --- | --- | --- | --- | --- | --- | --- |
| **Tangible/ instrumental support** | **Domestic** | Barrier | x | x | x | x | x | x | x | x |
|  |  | Enabler | Biswas 2017  Parveen 2018  Sultana 2018 | 5 | No concerns | Moderate concerns | Serious concerns | Serious concerns | Low | Number of studies limiting adequacy and geographical/population relevance (all Bangladesh) |
|  | **Institutional** | Barrier | x | x | x | x | x | x | x | x |
|  |  | Enabler | x | x | x | x | x | x | x | x |
|  | **Public** | Barrier | x | x | x | x | x | x | x | x |
|  |  | Enabler | x | x | x | x | x | x | x | x |
| **Network support** | **Domestic** | Barrier | Rahman 2017  Parveen 2018  White 2022 A | 5 | No concerns | Moderate concern | Serious concerns | Serious concerns | Low | Number of studies limiting adequacy and geographical/population relevance. |
|  |  | Enabler | Dearden 2002  Langford 2013  Rahman 2017  Scott 2007  Parveen 2018  Thaivalappil 2022  White 2022 A | 5 | No concerns | Minor concern | Minor concern | Minor concern | Moderate | Limited geographical relevance (mostly Africa, Asia). |
|  | **Institutional** | Barrier | x | x | x | x | x | x | x | x |
|  |  | Enabler | Green 2005  Schmidt 2009 | 5 | No concerns | Moderate concern | Serious concerns | Serious concerns | Very low | Number of studies limiting adequacy and geographical/population relevance. |
|  | **Public** | Barrier | x | x | x | x | x | x | x | x |
|  |  | Enabler | Watson 2020 | 5 | No concerns | Moderate concern | Serious concerns | Serious concerns | Very low | Number of studies limiting adequacy and geographical/population relevance. |

| **Summary of review finding** | **Setting** | **Barrier/ Enabler** | **Studies contributing to finding** | **Mean Theme**  **MMAT Score** | **Methodological limitations^1,2^ (Informed by  MMAT)**^3^ | **Coherence ^1,2^** | **Adequacy^, 1,2^** | **Relevance^, 1,2^** | **Overall GRADE-CERQual confidence assessment^4^** | **Explanation of GRADE-CERQual assessment** |
| --- | --- | --- | --- | --- | --- | --- | --- | --- | --- | --- |
| **Cultural practices and norms** | **Domestic** | Barrier | Aberese-Ako 2023  Hoque 2023  Kalumbi 2020  Parveen 2018  Ward 2022  White 2022 B | 4.8 | Very minor concerns | Minor concern | Minor concern | Moderate concern | Moderate | Limited geographical relevance (all Africa, Asia). |
|  |  | Enabler | Afolabi 2022  Chidziwisano 2019  Thaivalappil 2022  Zangana 2020 | 5 | No concerns | Moderate concern | Moderate concern | Serious concerns | Low | Limited geographical relevance (no Asia). |
|  | **Institutional** | Barrier | x | x | x | x | x | x | x | x |
|  |  | Enabler | Jackson 2021 | 5 | No concerns | Serious concern | Serious concerns | Serious concerns | Very low | Limited number of studies limiting adequacy and geographical/population relevance. |
|  | **Public** | Barrier | x | x | x | x | x | x | x | x |
|  |  | Enabler | x | x | x | x | x | x | x | x |

| **Summary of review finding** | **Setting** | **Barrier/ Enabler** | **Studies contributing to finding** | **Mean Theme**  **MMAT Score** | **Methodological limitations^1,2^ (Informed by  MMAT)**^3^ | **Coherence ^1,2^** | **Adequacy^, 1,2^** | **Relevance^, 1,2^** | **Overall GRADE-CERQual confidence assessment^4^** | **Explanation of GRADE-CERQual assessment** |
| --- | --- | --- | --- | --- | --- | --- | --- | --- | --- | --- |
| **MOTIVATION** | | | | | | | | | | |
| **Reflective Motivation** | | | | | | | | | | |
| **Perceived health risk** | **Domestic** | Barrier | Azam 2022  Parveen 2018  Tibbels 2022  Thaivalappil 2022 | 5 | No concerns | Moderate concerns | Moderate concerns | Serious concerns | Low | Number of studies limiting adequacy and geographical/population relevance. |
|  |  | Enabler | Aberese-Ako 2023  Affleck 2012  Afolabi 2022  Akter 2014  Curtis 2003  Dearden 2002  Biswas 2017  Didier 2021  Kalumbi 2020  Lanfer 2021  Langford 2013  Lohiniva 2007  Nizame 2013  Norrie 2022  Ogutu 2022  Rahman 2017  Scott 2007  Parveen 2018  Steiner-Asiedu 2011  Tibbels 2022  Torres-Slimming 2019  Ward 2022  White 2022 B  Zangana 2020 | 5 | No concerns | No concerns | No concerns | Minor concerns | High | Adequate number of studies supporting theme across multiple settings |

| **Summary of review finding** | **Setting** | **Barrier/ Enabler** | **Studies contributing to finding** | **Mean Theme**  **MMAT Score** | **Methodological limitations^1,2^ (Informed by  MMAT)**^3^ | **Coherence ^1,2^** | **Adequacy^, 1,2^** | **Relevance^, 1,2^** | **Overall GRADE-CERQual confidence assessment^4^** | **Explanation of GRADE-CERQual assessment** |
| --- | --- | --- | --- | --- | --- | --- | --- | --- | --- | --- |
| **Perceived health risk**  **(continued)** | **Institutional** | Barrier | Al-Naggar 2013  Sebong 2021 | 5 | No concerns | Serious concerns | Serious concerns | Serious concerns | Very low | Number of studies limiting adequacy and geographical/population relevance (Asia only; Universities only). |
|  |  | Enabler | Mbakaya 2019  Okello 2019  Pragle 2007  Schmidt 2009  Sebong 2021 | 5 | No concerns | Minor concerns | Minor concerns | Minor concerns | Moderate | Adequate number of studies supporting theme across multiple settings, though mostly schools in Asia/Africa. |
|  | **Public** | Barrier | x | x | x | x | x | x | x | x |
|  |  | Enabler | Babalobi 2013  Blum 2019  Watson 2020 | 5 | No concerns | Moderate concerns | Serious concerns | Serious concerns | Very low | Number of studies limiting coherence, adequacy, geographical/ population relevance (Africa/Asia only; IDP camps and market). |
| **Time Prioritization** | **Domestic** | Barrier | Affleck 2012  Akter 2014  Curtis 2003  Dearden 2002  Langford 2013  Lohiniva 2007  Nizame 2016  Rauyajin 1994  Sagan 2019  Parveen 2018  Steiner-Asiedu 2011 | 5 | No concerns | No concerns | No concerns | Minor concerns | High | Overall, adequate number of studies supporting theme across multiple settings. Limitation in geographic relevance (mostly Asia, specifically Bangladesh). |
|  |  | Enabler | Dearden 2002  Biswas 2017 | 5 | No concerns | Serious concerns | Serious concerns | Serious concerns | Very low | Number of studies limiting adequacy and geographical/population relevance. |

| **Summary of review finding** | **Setting** | **Barrier/ Enabler** | **Studies contributing to finding** | **Mean Theme**  **MMAT Score** | **Methodological limitations^1,2^ (Informed by  MMAT)**^3^ | **Coherence ^1,2^** | **Adequacy^, 1,2^** | **Relevance^, 1,2^** | **Overall GRADE-CERQual confidence assessment^4^** | **Explanation of GRADE-CERQual assessment** |
| --- | --- | --- | --- | --- | --- | --- | --- | --- | --- | --- |
| **Time Prioritization**  **(continued)** | **Institutional** | Barrier | Al-Naggar 2013  La Con 2017  Green 2005  Jackson 2021  Pragle 2007  Schmidt 2009  Steenkamp 2022 | 5 | No concerns | No concerns | No concerns | Minor concerns | High | Overall, adequate number of studies support theme across multiple settings. Limitation in geographic relevance though multiple regions and settings represented (though sometimes only once). |
|  |  | Enabler | x | x | x | x | x | x | x | x |
|  | **Public** | Barrier | Blum 2019  Mezaache 2021  Neetu 2013  Nizame 2019  Wu 2019 | 4.4 | Minor concerns | Minor concerns | Minor concerns | Minor concerns | Moderate | Limitation in geographic relevance (mostly Asia, Africa); though multiple settings represented (e.g., Markets, parks, harm reduction centers).). |
|  |  | Enabler | x | x | x | x | x | x | x | x |
| **Water Prioritization** | **Domestic** | Barrier | Lohiniva 2007  Mshida 2020  Ogutu 2022  Thorseth 2021 | 5 | No concerns | No concerns | Moderate concerns | Moderate concerns | Moderate | Limitation in geographic relevance (all rural Africa). |
|  |  | Enabler | x | x | x | x | x | x | x | x |
|  | **Institutional** | Barrier | x | x | x | x | x | x | x | x |
|  |  | Enabler | x | x | x | x | x | x | x | x |
|  | **Public** | Barrier | x | x | x | x | x | x | x | x |
|  |  | Enabler | x | x | x | x | x | x | x | x |

| **Summary of review finding** | **Setting** | **Barrier/ Enabler** | **Studies contributing to finding** | **Mean Theme**  **MMAT Score** | **Methodological limitations^1,2^ (Informed by  MMAT)**^3^ | **Coherence ^1,2^** | **Adequacy^, 1,2^** | **Relevance^, 1,2^** | **Overall GRADE-CERQual confidence assessment^4^** | **Explanation of GRADE-CERQual assessment** |
| --- | --- | --- | --- | --- | --- | --- | --- | --- | --- | --- |
| **Willingness to practice hand- washing** | **Domestic** | Barrier | Affleck 2012  Didier 2021  Herbst 2009  Greenwell 2013  Mohamed 2022  Rauyajin 1994  Parveen 2018 | 4.7 | Very minor concerns | Moderate concerns | Moderate concerns | Minor concerns | Moderate | Overall, adequate number of studies supporting theme across multiple settings, though variability within theme across settings limiting coherence. |
|  |  | Enabler | Akter 2014  Dearden 2002  Harrison 2019  Herbst 2009  Rahman 2017  Parveen 2018 | 5 | No concerns | Moderate concerns | Moderate concerns | Minor concerns | Moderate | Overall, adequate number of studies supporting theme across multiple settings, though variability within theme across settings limiting coherence. |
|  | **Institutional** | Barrier | Al-Naggar 2013  Green 2005 | 5 | No concerns | Moderate concerns | Serious concerns | Serious concerns | Very low | Number of studies limiting adequacy and geographical/population relevance. |
|  |  | Enabler | Pragle 2007 | 5 | No concerns | Moderate concerns | Serious concerns | Serious concerns | Very low | Number of studies limiting adequacy and geographical/population relevance. |
|  | **Public** | Barrier | Thorseth 2021 | 5 | No concerns | Moderate concerns | Serious concerns | Serious concerns | Very low | Number of studies limiting adequacy and geographical/population relevance. |
|  |  | Enabler | x | x | x | x | x | x | x | x |

| **Summary of review finding** | **Setting** | **Barrier/ Enabler** | **Studies contributing to finding** | **Mean Theme**  **MMAT Score** | **Methodological limitations^1,2^ (Informed by  MMAT)**^3^ | **Coherence ^1,2^** | **Adequacy^, 1,2^** | **Relevance^, 1,2^** | **Overall GRADE-CERQual confidence assessment^4^** | **Explanation of GRADE-CERQual assessment** |
| --- | --- | --- | --- | --- | --- | --- | --- | --- | --- | --- |
| **Perceived ease or difficulty of washing hands** | **Domestic** | Barrier | Dearden 2002  Norrie 2022  Ward 2022  White 2022 A  Yardley 2011 | 5 | No concerns | Moderate concerns | Moderate concerns | Minor concerns | Moderate | Overall, somewhat limited number of studies supporting theme across multiple settings, and variability within theme across settings limiting coherence. Number limiting adequacy and relevance. |
|  |  | Enabler | Biswas 2017  Langford 2013  Mohamed 2022  Simiyu 2020  Sultana 2018  Ward 2022  White 2022 A | 4.7 | Very minor concerns | Moderate concerns | Minor concerns | Moderate concerns | Moderate | Overall, variability within theme across settings limiting coherence. Relevance limiting (All Africa, Asia). |
|  | **Institutional** | Barrier | Steenkamp 2022 | 5 | No concerns | Moderate concerns | Serious concerns | Serious concerns | Very low | Number of studies limiting coherence, adequacy, and geographical/population relevance. |
|  |  | Enabler | Randle 2013 | 5 | No concerns | Moderate concerns | Serious concerns | Serious concerns | Very low | Number of studies limiting coherence, adequacy, and geographical/population relevance. |
|  | **Public** | Barrier | Mezaache 2021  Nizame 2019  Wu 2019 | 4 | Minor concerns | Moderate concerns | Serious concerns | Serious concerns | Very low | Number of studies limiting coherence, adequacy, and geographical/population relevance. |
|  |  | Enabler | Mezaache 2021 | 2 | Serious concerns | Moderate concerns | Serious concerns | Serious concerns | Very low | Number of studies limiting coherence, adequacy, and geographical/population relevance. |

| **Summary of review finding** | **Setting** | **Barrier/ Enabler** | **Studies contributing to finding** | **Mean Theme**  **MMAT Score** | **Methodological limitations^1,2^ (Informed by  MMAT)**^3^ | **Coherence ^1,2^** | **Adequacy^, 1,2^** | **Relevance^, 1,2^** | **Overall GRADE-CERQual confidence assessment^4^** | **Explanation of GRADE-CERQual assessment** |
| --- | --- | --- | --- | --- | --- | --- | --- | --- | --- | --- |
| **Influence of religion** | **Domestic** | Barrier | Azam 2022  Sagan 2019  Tibbels 2022 | 5 | No concerns | Moderate concerns | Serious concerns | Serious concerns | Very low | Number of studies limiting coherence, adequacy, and geographical/population relevance. |
|  |  | Enabler | Afolabi 2022 | 5 | No concerns | Serious concerns | Serious concerns | Serious concerns | Very low | Number of studies limiting coherence, adequacy, and geographical/population relevance. |
|  | **Institutional** | Barrier | x | x | x | x | x | x | x | x |
|  |  | Enabler | x | x | x | x | x | x | x | x |
|  | **Public** | Barrier | x | x | x | x | x | x | x | x |
|  |  | Enabler | x | x | x | x | x | x | x | x |
| **Automatic Motivation** | | | | | | | | | | |
| **Internal motivation/ habit** | **Domestic** | Barrier | Aberese-Ako 2023  Affleck 2012  Akter 2014  Atuyambe 2011  Azam 2022  Curtis 2003  Dearden 2002  Didier 2021  Kalumbi 2020  Langford 2013  Lohiniva 2007  Nizame 2013  Nizame 2016  Rauyajin 1994  Parveen 2018  Thaivalappil 2022  Tibbels 2022 | 5 | No concerns | Minor concern | No concern | Minor concern | High | Overall, adequate number of studies supporting theme across multiple settings. |

| **Summary of review finding** | **Setting** | **Barrier/ Enabler** | **Studies contributing to finding** | **Mean Theme**  **MMAT Score** | **Methodological limitations^1,2^ (Informed by  MMAT)**^3^ | **Coherence ^1,2^** | **Adequacy^, 1,2^** | **Relevance^, 1,2^** | **Overall GRADE-CERQual confidence assessment^4^** | **Explanation of GRADE-CERQual assessment** |
| --- | --- | --- | --- | --- | --- | --- | --- | --- | --- | --- |
| **Internal motivation/ habit** | Domestic | Barrier | Aberese-Ako 2023  Affleck 2012  Akter 2014  Atuyambe 2011  Azam 2022  Curtis 2003  Dearden 2002  Didier 2021  Kalumbi 2020  Langford 2013  Lohiniva 2007  Nizame 2013  Nizame 2016  Rauyajin 1994  Parveen 2018  Thaivalappil 2022  Tibbels 2022 | 5 | No concerns | Minor concern | No concern | Minor concern | High | Overall, adequate number of studies supporting theme across multiple settings. |
|  |  | Enabler | Affleck 2012  Bauza 2021  Biran 2005  Chidziwisano 2019  Curtis 2003  Dearden 2002  Biswas 2017  Didier 2021  Greenwell 2013  Kalumbi 2020  Lando 2018  Langford 2013  Mitchell 2021  Sagan 2019  Sedekia 2022  Parveen 2018  Sultana 2018  Thaivalappil 2022  Yardley 2011 | 5 | No concerns | No concerns | No concerns | Minor concerns | High | Overall, adequate number of studies supporting theme across multiple settings. |

| **Summary of review finding** | **Setting** | **Barrier/ Enabler** | **Studies contributing to finding** | **Mean Theme**  **MMAT Score** | **Methodological limitations^1,2^ (Informed by  MMAT)**^3^ | **Coherence ^1,2^** | **Adequacy^, 1,2^** | **Relevance^, 1,2^** | **Overall GRADE-CERQual confidence assessment^4^** | **Explanation of GRADE-CERQual assessment** |
| --- | --- | --- | --- | --- | --- | --- | --- | --- | --- | --- |
| **Internal motivation/ habit**  **(continued)** | Institutional | Barrier | Al-Naggar 2013  Kumar 2018  Schmidt 2009 | 5 | No concerns | Moderate concerns | Serious concerns | Serious concerns | Low | Number of studies limiting adequacy and geographical/population relevance (mostly educational settings) |
|  |  | Enabler | Arendt 2015  Devkota 2020  Jackson 2021  Neetu 2013  Okello 2019  Pragle 2007 | 5 | No concerns | Moderate concerns | Moderate concerns | Moderate concerns | Moderate | Theme represented coherently and adequately though geographical/population relevance limited. |
|  | Public | Barrier | Blum 2019  Mezaache 2021  Thorseth 2021  Wu 2019 | 4.2 | Minor concerns | Moderate concerns | Moderate concerns | Moderate concerns | Low | Number of studies limiting adequacy and geographical/population relevance |
|  |  | Enabler | x | x | x | x | x | x | x | x |
| **Feeling of cleanliness** | **Domestic** | Barrier | x | x | x | x | x | x | x | x |
|  |  | Enabler | Afolabi 2022  Biran 2005  Curtis 2003  Demberere 2016  Ogutu 2022  Scott 2007  Parveen 2018  White 2022 A Zangana 2020 | 5 | No concerns | Minor concerns | Minor concerns | Minor concerns | High | Overall, adequate number of studies supporting theme across multiple settings. |
|  | **Institutional** | Barrier | Schmidt 2009 | 5 | No concerns | Serious concerns | Serious concerns | Serious concerns | Very low | Number of studies limiting adequacy and geographical/population relevance (UK school only) |
|  |  | Enabler | Schmidt 2009 | 5 | No concerns | Serious concerns | Serious concerns | Serious concerns | Very low | Number of studies limiting adequacy and geographical/population relevance (UK school only) |

| **Summary of review finding** | **Setting** | **Barrier/ Enabler** | **Studies contributing to finding** | **Mean Theme**  **MMAT Score** | **Methodological limitations^1,2^ (Informed by  MMAT)**^3^ | **Coherence ^1,2^** | **Adequacy^, 1,2^** | **Relevance^, 1,2^** | **Overall GRADE-CERQual confidence assessment^4^** | **Explanation of GRADE-CERQual assessment** |
| --- | --- | --- | --- | --- | --- | --- | --- | --- | --- | --- |
| **Feeling of cleanliness**  **(continued)** | **Public** | Barrier | x | x | x | x | x | x | x | x |
|  |  | Enabler | Blum 2019  Mezaache 2021  Neetu 2013  Wu 2019 | 4.2 | Minor concerns | Serious concerns | Serious concerns | Moderate concerns | Low | Number of studies limiting adequacy and geographical/population relevance (varied countries and locations, but ideas not saturated) |
| **Like/ dislike of handwash product** | **Domestic** | Barrier | Dearden 2002  Ward 2022 | 5 | No concerns | Moderate concnern | Serious concerns | Serious concerns | Very low | Number of studies limiting adequacy and geographical/population relevance |
|  |  | Enabler | Biran 2005  Langford 2013 | 5 | No concerns | Serious concerns | Serious concerns | Serious concerns | Very low | Number of studies limiting adequacy and geographical/population relevance |
|  | **Institutional** | Barrier | x | x | x | x | x | x | x | x |
|  |  | Enabler | Randle 2013  Schmidt 2009 | 5 | No concerns | Serious concerns | Serious concerns | Serious concerns | Very low | Number of studies limiting adequacy and geographical/population relevance |
|  | **Public** | Barrier | Mezaache 2021 | 2 | Serious concerns | Serious concerns | Serious concerns | Serious concerns | Very low | Number of studies limiting adequacy and geographical/population relevance |
|  |  | Enabler | Blum 2019  Mezaache 2021  Wu 2019 | 4 | Minor concerns | Moderate concerns | Moderate concerns | Serious concerns | Low | Number of studies limiting adequacy and geographical/population relevance |
| 1. Definitions from: Lewin S, Booth A, Glenton C, Munthe-Kaas H, Rashidian A, Wainwright M, Bohren MA, Tunçalp Ö, Colvin CJ, Garside R, Carlsen B. Applying GRADE-CERQual to qualitative evidence synthesis findings: introduction to the series. Implementation Science. 2018 Jan;13:1-0.  2. No or very minor concerns / minor concerns / moderate concerns / serious concerns  3. See supplemental table 8 for MMAT scoring and further information about how overall MMAT scores were generated. For more information on MMAT see: Hong QN, Pluye P, Fàbregues S, et al. Mixed methods appraisal tool (MMAT) version 2018: user guide. Montr McGill Univ 2018; 1.  4. Is this synthesis finding "a reasonable representation of the phenomenon of interest?". Score categories: high, moderate, low, very low | | | | | | | | | | |
|  | | | | | | | | | | |
